# Genome-guided comparative *in planta* transcriptome analyses for identifying cross-species common virulence factors in bacterial phytopathogens

**DOI:** 10.3389/fpls.2022.1030720

**Published:** 2022-11-16

**Authors:** Jungwook Park, Hyejung Jung, Mohamed Mannaa, Seung Yeup Lee, Hyun-Hee Lee, Namgyu Kim, Gil Han, Dong-Soo Park, Sang-Won Lee, Seon-Woo Lee, Young-Su Seo

**Affiliations:** ^1^ Department of Integrated Biological Science, Pusan National University, Busan, South Korea; ^2^ Biotechnology Research Division, National Institute of Fisheries Science, Busan, South Korea; ^3^ Department of Applied Bioscience, Dong-A University, Busan, South Korea; ^4^ Paddy Crop Division, National Institute of Crop Science, Rural Development Administration, Miryang, South Korea; ^5^ Department of Plant Molecular Systems Biotech & Crop Biotech Institute, KyungHee University, Yongin, South Korea

**Keywords:** virulence, comparative *in planta* transcriptomic, genome, phytopathogenic bacteria, rice

## Abstract

Plant bacterial disease is a complex outcome achieved through a combination of virulence factors that are activated during infection. However, the common virulence factors across diverse plant pathogens are largely uncharacterized. Here, we established a pan-genome shared across the following plant pathogens: *Burkholderia glumae*, *Ralstonia solanacearum*, and *Xanthomonas oryzae* pv. *oryzae*. By overlaying *in planta* transcriptomes onto the pan-genome, we investigated the expression profiles of common genes during infection. We found over 70% of identical patterns for genes commonly expressed by the pathogens in different plant hosts or infection sites. Co-expression patterns revealed the activation of a signal transduction cascade to recognize and respond to external changes within hosts. Using mutagenesis, we uncovered a relationship between bacterial virulence and functions highly conserved and shared in the studied genomes of the bacterial phytopathogens, including flagellar biosynthesis protein, C4-dicarboxylate ABC transporter, 2-methylisocitrate lyase, and protocatechuate 3,4-dioxygenase (PCD). In particular, the disruption of PCD gene led to attenuated virulence in all pathogens and significantly affected phytotoxin production in *B. glumae*. This PCD gene was ubiquitously distributed in most plant pathogens with high homology. In conclusion, our results provide cross-species *in planta* models for identifying common virulence factors, which can be useful for the protection of crops against diverse pathogens.

## 1 Introduction

Plant pathogenic bacteria cause fatal diseases in their hosts, leading to reduced agricultural productivity, which can eventually result in massive economic losses. Of these plant pathogenic bacteria, most belong to the following genera within the phylum Proteobacteria: *Pseudomonas*, *Ralstonia*, *Burkholderia*, *Agrobacterium*, *Xanthomonas*, and *Pectobacterium*. Within a distinct environment of hosts, the pathogenicity of plant pathogens is a complex activity achieved through a myriad of virulence factors that enable the acquisition of resources, overcoming of host defenses, and production of toxic molecules (Kim et al., 2004; Gudesblat et al., 2009; Dalsing et al., 2015). Until now, most studies have been limited to the functional validation of only one or a few virulence factors, in a single pathogen, as opposed to revealing an entire landscape of plant pathogenesis or common virulence factors across phytopathogenic species (Sundin et al., 2016). However, to develop practical strategies for the control of bacterial diseases, the convergence and/or divergence of virulence mechanisms should be fully understood. Advances in next-generation sequencing technology have accelerated comparative genomic studies thanks to the elucidation of the genomes of various organisms. The genomic architecture of pathogens is an important resource of adaptive evolution for specialized lifestyles in each host niche (Jones et al., 2021). However, only genome-dependent analyses may not define individual characteristics among pathogens due to the deviation of unique gene repertoires by the population size. It is also difficult to consider the importance of regulatory genes that affect multiple systems, including infection mechanisms or adaptation processes within plant host tissues. Hence, a universal model based on the expression patterns will help to elucidate the complexity of global plant-pathogen interactions and allow for the identification of novel virulence factors.

Comparative analysis of *in planta* transcriptomes across plant pathogens may be one of the best solutions to fill this knowledge gap. *In planta* transcriptome analysis involves monitoring the gene expression profiles of pathogens during infection. The genome-wide modification of transcriptome during *in planta* infection is the inevitable outcome of bacterial adaptation to a hostile environment (Yadeta and Thomma, 2013). Although several studies have investigated the transcriptome of pathogens, either in plant-mimicking media or in whole plant extracts (Ciraulo et al., 2010; Yazawa et al., 2013), the quality or quantity of the RNAs only partially reflect the *in planta* conditions. Obtaining a high-quality transcriptome of pathogenic bacteria directly from the infected tissues is a crucial step in any such analysis (Azhikina et al., 2010; Skvortsov and Azhikina, 2010). Some previous studies reported high-quality *in planta* transcriptomes of plant pathogenic bacteria (Jacobs et al., 2012; Kim et al., 2014).

As a destructive pathogenic bacterium, *Ralstonia solanacearum* causes bacterial wilt in most solanaceous plants, including tomato, tobacco, and potato (Gerlin et al., 2021; Nion and Toyota, 2015). Jacobs et al. (2012), characterized the *in planta* transcriptome of *R. solanacearum* GMI1000 within tomato stems. Another extensively studied plant pathogen is *Burkholderia glumae*, which causes grain and seedling rot in rice and bacterial wilt in many field crops, including tomato, eggplant, and pepper (Ham et al., 2011; Riera-Ruiz et al., 2014). We previously reported the *in planta* transcriptional profiles of *B. glumae* BGR1 in rice stems (Kim et al., 2014). A comparison of independently acquired *in planta* transcriptomes with *in vitro* transcriptomes revealed substantial changes in the expression profiles of genes involved in various metabolic and signaling pathways in both *R. solanacearum* and *B. glumae* (Jacobs et al., 2012; Kim et al., 2014). In the present study, to increase the number of available bacterial transcriptomes, we constructed *in planta* transcriptomes of *Xanthomonas oryzae* pv. *Oryzae* (*Xoo*) KACC10331 within rice leaves, using high-throughput RNA sequencing (RNA-seq). *Xoo* is the causative agent of bacterial leaf blight, which is responsible for losses in rice yield of up to 50% worldwide (Niño-Liu et al., 2006).

Therefore, expanding our understanding of the transcriptomes of plant pathogenic *B. glumae*, *R. solanacearum*, and *Xoo* will support the biological interpretation of pathogenicity and provide a valuable reference for comparative analyses involving multi-species of phytopathogenic bacteria. Given a sessile and autotrophic lifestyle that limits resource availability, land plants face a fundamental dilemma: either efficiently allocate limited resources to optimal growth or economically invest these resources in defense strategies against invaders (Pageau et al., 2006; Schultz et al., 2013; Veillet et al., 2016). Unlike the immune system of animals, plant immunity consists of broad and slightly less complex strategies that have developed through evolution and are shared among plant species (Tao et al., 2003; Maekawa et al., 2011). Accordingly, comparing the *in planta* transcriptomes of different pathogens enables the identification of fundamental principles for their survival in response to integrated plant conditions that transcend different hosts, infection sites, or environments. In addition, *B. glumae* shares a broad host range with *Xoo* in rice and with *R. solanacearum* in vegetable crops. *B. glumae* can attack diverse infection sites, including grains, stems, and seedlings. Similar to the other two pathogens, it can also infect vascular systems. The genome organization of *B. glumae* shows a high degree of plasticity to cope with such a variety of environments (Seo et al., 2015). From this perspective, the genomic information of *B. glumae* can be applied as a central guide to identify the common factors employed by three plant pathogens.

This study was conducted to compare the *in planta* transcriptomes across three major bacterial phytopathogens in an attempt to infer a universal model of their dynamic interactions with plant hosts. A pan-genome guided map was utilized to characterize the highly conserved *in planta* co-expression patterns among the three phytopathogens. In addition, we aim to identify the key functions of genes comprising the common virulence of the genomes of studied species and validate their involvement in virulence through mutagenesis experiments. The provided methodology represents an applicable strategy for investigating *in planta* interactions across plant pathogens and identifying common virulence factors expressed during infection.

## 2 Materials and methods

### 2.1 Pan-genome analysis

Data for comparative genome and transcriptome analyses were obtained from the NCBI genome server (ftp://ftp.ncbi.nlm.nih.gov/genomes/) and the GEO database (http://www.ncbi.nlm.nih.gov/geo/), as described in the Supporting Information.

The BRIG tool was used to provide the relationships among the whole genome sequences of the three pathogens (http://brig.sourceforge.net/). All genome replicons of *B. glumae* BGR1 were selected to form a reference sequence. The genome sequences of *R. solanacearum* GMI1000 and *Xoo* KACC10331 were aligned to the reference using the BLASTn method, with the following parameters: upper identity, 70%; lower identity, 50%; and E-value, 0.001. All results were displayed as a series of concentric rings, with the central ring representing the reference genome. Subsequently, a progressive Mauve algorithm with default parameters was used to investigate genome rearrangements (http://darlinglab.org/mauve/).

For pan-genome analysis, a BLASTp search was used to align the sequences of all genes of *B. glumae* to the reference *R. solanacearum* and *Xoo* genomes. To exclude random hits, alignments were filtered according to the significance criteria of 50% coverage, 50% identity, and an E-value of 1.0 × 10^-5^, as previously described (Anderson and Brass, 1998). After sorting according to the E-value, the best-matched subject from all results per query was selected and re-aligned against the reference *B. glumae* genome to identify complete gene sets with a high sequence correlation. To investigate the unique genomes, unmatched *R. solanacearum* and *Xoo* genes were compared with each other. The genes in the pan-genome map were analyzed and subdivided into orthologous and non-orthologous groups. The COGs database was used to analyze the functional details of each group (http://www.ncbi.nlm.nih.gov/research/cog-project/). Amino acid sequences were scanned against COG categories using BLASTp, with a cut-off of 50% coverage, 50% identity, and an E-value of 1.0 × 10^-5^.

### 2.2 Comparative *in planta* transcriptome analysis

The *in planta* transcriptomes of *Xoo* KACC10331 were constructed, the methodological details of which are provided in the Supporting Information. Raw data generated in this study have been submitted to the GEO database under accession number GSE89651.

Raw reads from the RNA-seq library were filtered for a minimum Phred score of 28 in >50% lengths using the FASTX-Toolkit (http://hannonlab.cshl.edu/fastx_toolkit/). The BWA-MEM algorithm was used to align pre-processed reads to the reference genome of each pathogen (http://bio-bwa.sourceforge.net/). SAM files derived from mapping were converted to binary format, and then sorted by chromosomal coordinates using the SAMtools (http://www.htslib.org/). The read counts of the annotated genes were normalized as reads per kilobase of transcripts per million mapped reads. To identify DEGs, the DEseq package with a MA plot-based method was implemented in R (Wang et al., 2010). The Z-score of two samples was estimated and converted to a two-sided *P*-value, which was used to establish whether a gene was differentially expressed or not. For microarray analysis, raw data were analyzed following standard methods, as previously described (Jacobs et al., 2012). A total of eight *in planta* and *in vitro* libraries were normalized using the quantile normalization method. The Student’s *t*-test was used to calculate *P*-values denoting differences between two samples. In both RNA-seq and microarray analyses, a false discovery rate (FDR) was calculated to control the error rate for multiple comparisons. Finally, the same criteria of FDR < 0.05 and log_2_(*in planta*/*in vitro*) ≥ 1 were used for DEG identification.

All DEGs were classified into orthologous and non-orthologous groups in the guided pan-genome map. The expression levels of classified DEGs were compared using the heatmap and 3D scatterplot to investigate tendencies in the expression patterns. To reveal the biological significance of co-expression patterns, DEGs that followed identical tendencies among plant pathogens were subjected to GO enrichment analysis. The GO terms were used to interpret functional sets of genes classified according to the associated biological process, molecular function, and cellular component. The entire GO background was downloaded in the UniProt database (http://www.uniprot.org/), while specific GO terms were identified based on upregulated and downregulated DEGs. Incomplete GO terms which matched <5 genes were excluded from the analysis. In the unique genomes, *in planta*-dependent DEGs were evaluated by enrichment analysis of biological pathways obtained from the KEGG database (http://www.genome.jp/kegg/). A hypergeometric distribution was used to perform statistical enrichment with *P* ≤ 0.05, as described in the Supporting Information. Subsequently, enriched pathways in the three pathogens were compared to uncover unique features. The genes associated with the uniquely upregulated pathways were mapped against all biological reactions using the KEGG Mapper. The Cytoscape tool was used for the construction of molecular networks with nodes as genes and edges as interactions (http://cytoscape.org/).

### 2.3 Bacterial virulence assays

#### 2.3.1 Plant assay for testing the virulence of *B. glumae* and the four constructed mutants

To evaluate the effect of the four shared key functional genes on virulence, a single colony inoculation assay was performed using the constructed *B. glumae* mutants according to the method described by Kim et al. (2019). The wild-type strain BGR1 and four mutants were incubated at 37°C on LB agar plates until the diameter of single colonies reached approximately 1 mm. Rice plants (cv. Dongjin) were grown in a greenhouse before and after inoculation with *B. glumae* strains. At the vegetative stage, rice stems were scratched with a syringe needle(scratch diameter; ~1 mm), following which a single colony per strain was applied using a sterile micropipette tip. The infection court at the inoculated stems was observed seven days later. Photographs of diseased rice stems including inoculation sites were acquired. The diseased stem area (%) was recorded to facilitate a more accurate assessment of the effect of virulence through mutations. Using the Adobe Photoshop CS6 with the color range module (Adobe, San Jose, CA, USA), the pixel size of infection court was measured, and the diseased ratio was calculated by comparing the entire size of inoculated rice stem. Differences in disease symptoms were analyzed and plants that did not undergo inoculation served as a negative control.

#### 2.3.2 Plant assays to confirm the influence of PCD-disruption on virulence of the three bacterial species

Representative virulence assays for the three pathogens were carried out using PCD-disrupted mutants. The methodological details of the mutagenesis experiments are included in the Supporting Information. The virulence of *R. solanacearum* strains was assessed by inoculating 3–4-week-old tomato plants (cv. Zuiken) by natural soil-soaking inoculation (Tans-Kersten et al., 2004). Bacterial cells were grown in casamino acid peptone glucose medium and washed twice with distilled water. For bacterial inoculation, each tomato plant was exposed to a bacterial suspension at a final OD_600_ of approximately 0.01 per gram of soil. All inoculations included 10 tomato plants for each strain. The inoculated plants were incubated in a growth chamber under light for 14 h and in the dark for 10 h at 28°C. For 15 days after inoculation, disease progression was rated using the following scale: 0, no wilting; 1, 1–25% wilting; 2, 25–50% wilting; 3, 51–75% wilting; and 4, 76–100% wilted or dead.

For the leaf-clipping virulence assay using *Xoo*, rice plants (cv. Dongjin) were grown in the greenhouse until the vegetative stage (5–6 weeks). Each strain was cultured on a peptone sucrose (PS) medium at 28°C with shaking at 200 rpm, and its concentration was adjusted in sterile distilled water to OD_600_ = 0.8. The bacterial suspensions were clip-inoculated on rice leaves using scissors. The lesion lengths of the clipped leaves were measured 14 days after inoculation.

For the panicle blight assay, it was conducted as described previously (Kim et al., 2019), using *B. glumae*, as rice plants (cv. Dongjin) were inoculated at the flowering stage with a bacterial suspension (OD_600_ = 0.5) and grown in a greenhouse. Disease in the rice panicles was evaluated after 14 days, using the following scale: 0, healthy; 1, 0–20% discolored; 2, 20–40% discolored; 3, 40–60% discolored; 4, 60–80% discolored; and 5, 80–100% discolored. The disease index was determined using the following equation: ∑ (number of samples per score × score)/total number of panicles. For all virulence assays, plants that did not undergo inoculation served as negative controls

### 2.4 Mutant growth assay under the protocatechuic acid condition

To evaluate the major physiological changes of the PCD-disrupted mutant of *B. glumae* under PCA treatment, cultured cells were subcultured in 3 ml of fresh Luria-Bertani (LB) medium supplemented with 1 mg/ml PCA, as previously described (Stojković et al., 2013). Bacterial growth in terms of OD_600_ was measured every 2 h using a UV-1800 spectrophotometer (Shimadzu, Kyoto, Japan). For the spot dilution assay, overnight cultures of each strain were subcultured in a fresh medium until reaching an OD_600_ of 0.5. Next, 1:10 serial dilutions were prepared in fresh LB broth. Each culture (10 μl) was spotted on LB agar plates containing the same concentration of PCA as above. After incubation at 37°C for 24 h, colony formation was photographed and the number of colonies was counted to determine the viability of bacterial cells. Results were expressed as log(CFU/ml) per sample of three experiments.

### 2.5 Statistical analysis

Statistical analyses were carried out in the R v3.6.3 environment and Statistical Analysis Systems (SAS Institute, Cary, NC). The methods used for statistical analysis of bioinformatics data are mentioned directly in the corresponding section in the Materials and Methods. At least three biological replicates were used for each experiment. For the plant assays, disease severity data from repeated experiments were pooled together after confirmation of the homogeneity of variance. Analysis of variance was performed using the GLM procedure, and the means were separated using the least significant difference test at *P* < 0.05.

## 3 Results

### 3.1 Genome comparison reveals highly conserved regions among three pathogens

The genomic information of the three pathogenic bacteria is summarized in **Table S1**. As members of the Burkholderiaceae family of the class β-proteobacteria, *B. glumae* BGR1 and *R. solanacearum* GMI1000 display similar chromosomal replicons, in terms of size (3.91 and 3.72 Mb, respectively), GC content (68.1% and 67.0%, respectively), and gene count (3568 and 3429 genes, respectively). In contrast, the genome of *Xoo* KACC10331, a member of γ-proteobacteria, had only one chromosome with the highest number of genes (4568 genes).

**Figure 1:**
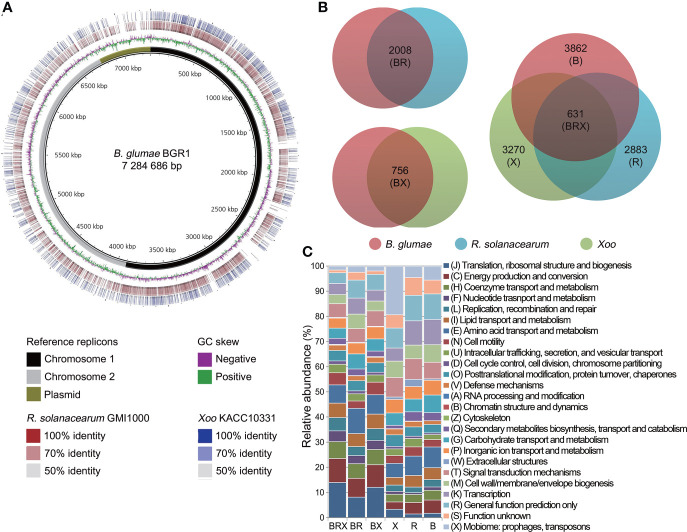
Comparative genome analysis among three pathogens **(A)** Multiple genome comparisons were performed using the **(*B*)** *glumae* BGR1 genome as a reference. The two innermost circles represent the reference sequence and GC skew, respectively. The reference sequence of **(*B*)** *glumae* BGR1 included three genome replicons (black, chromosome 1; grey, chromosome 2; and olive, plasmids) combined into a single contig. The outer rings illustrate the similarity of the *R. solanacearum* GMI1000 and *Xoo* KACC10331 genomes to the reference sequence. Higher color intensity represents a higher percentage of identity. **(B)** Venn diagram illustrating the sizes of orthologous and non-orthologous groups in the pan-genome. The genetic features of each pathogen are grouped within colored circles as follows: BR group between **(*B*)** *glumae* and *R. solanacearum*; BX group between **(*B*)** *glumae* and *Xoo*; BRX group among the three pathogens; and B, R, and X non-orthologous groups of each pathogen. **(C)** Frequencies of genes within 25 COG categories are illustrated for the orthologous and non-orthologous groups. The bar chart indicates the percentage of genes belonging to each functional category relative to the total number of genes in all COG categories. COG categories are displayed on the right side of the chart.

The reference genome of *B. glumae* BGR1 was compared with that of *R. solanacearum* GMI1000 and *Xoo* KACC10331 to define their relationship at the genomic level (**Figure 1A**). Consistent with genome organization, *R. solanacearum* was closely related to *B. glumae*, with 9131 homologous matches. However, *Xoo* also shared many homologous regions (3609 matches) with *B. glumae*. Among these, 60.18% (2172 matches) showed over 70% identity, and many aligned regions overlapped with those shared with *R. solanacearum*. Furthermore, the genome rearrangements of chromosome 1 replicons, which carried the highest number of genes among genome replicons, were investigated (**Figure S1**). This analysis revealed that 58 locally collinear blocks (LCBs) were shared by *B. glumae* and *R. solanacearum*. Despite rearrangements and inversions, the comparison between *B. glumae* and *Xoo* also showed many conserved regions in 53 LCBs.

To identify the biological functions conserved in the three pathogens, a pan-genome map was constructed from the comparison matrix relative to *B. glumae* and the unique genomes of each bacterium (**Figure 1B**). The comparison of *B. glumae* with *R. solanacearum* revealed a large orthologous genome (BR group) that included 2008 genes. The BX orthologous group, consisting of 756 genes, was found to be shared by *B. glumae* and *Xoo*. Of these, 631 genes (83.47%) were found to be shared in the three studied genomes (BRX group) of the three pathogens, *B. glumae*, *R. solanacearum* and *Xoo.*

Furthermore, 3862, 2883, and 3270 genes were unique to the B, R, and X groups, respectively. All genes in each group were annotated with 25 cluster of orthologous groups (COGs) functional categories (**Figure 1C**; **Table S2**). In the orthologous groups, “translation, ribosomal structure, and biogenesis” represented the largest category, including an average of 11.38% of the genes, followed by “energy production and conversion” (8.66%). In contrast, the “mobilome: prophages, transposons” (9.86%) and “function unknown” (5.93%) categories were prominent in the non-orthologous groups.

### 3.2 Co-expression patterns are associated with recognition and response to plant hosts

In *Xoo* KACC10331, six RNA-seq libraries were sequenced to generate 17,833,318 to 29,295,580 paired-end reads. All pre-processing and mapping data were of sufficient quantity and quality to ensure transcriptome accuracy (**Table S3**). The comparison of *in planta* samples with *in vitro* samples allowed for the identification of 1383 upregulated and 710 downregulated differentially expressed genes (DEGs) during infection (**Figure S2**). Subsequently, *in planta* transcriptome analyses of *B. glumae* and *R. solanacearum* were carried out independently. Consequently, 2405 and 890 *in planta*-dependent DEGs were identified in *B. glumae* and *R. solanacearum*, respectively (**Figure S2**). The DEGs of each pathogen represented a substantial fraction of the gene pool.

**Figure 2:**
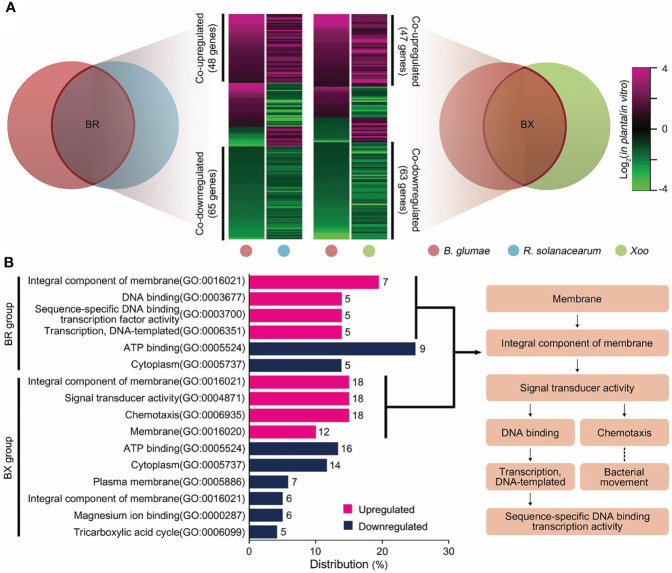
Co-expression patterns of in planta transcriptomes in the three pathogens **(A)** Expression patterns from pairwise orthologous groups are shown as heatmaps. Each column represents a pathogen, and each row represents a DEG. The relative expression level of DEGs is presented using a fold change value, shown as log_2_(*in planta*/*in vitro*). Red and green gradients indicate an increase or decrease in gene expression, respectively. Co-expression patterns that followed identical tendencies among plant pathogens are shown on the *y*-axis with their gene scale. **(B)** Co-upregulated (red) and co-downregulated (blue) DEGs were subjected to GO analysis. GO terms in the upper and lower parts of the bar chart are enriched in the BR and BX orthologous groups, respectively. The *x*-axis shows the distribution of each term with gene count among all GO terms. The flowchart illustrates the cascade of co-upregulated GO terms associated with signal transduction considered in our prediction model.

To explore co-expression patterns among *in planta* transcriptomes, DEGs were matched to the orthologous groups of the guided pan-genome map. Pairwise comparisons with the BR and BX groups revealed 158 and 146 common DEGs, respectively (**Figure 2A**); over 70% of DEGs showed identical expression patterns (**Table S4**). In the BR group, 113 genes formed co-expression patterns with 48 upregulated and 65 downregulated DEGs. Moreover, 47 co-upregulated and 63 co-downregulated DEGs displayed identical tendencies in the BX group. Then, gene ontology (GO) analysis was performed to understand the biological significance of co-expression patterns (**Figure 2B**). Interestingly, the functions of co-upregulated DEGs could be linked to form a cascade of signaling mechanisms from the recognition of external stimuli: “integral component of membrane” → “signal transducer activity” → “chemotaxis”, or “sequence-specific DNA binding transcription activity”. The function “chemotaxis” represents one such signaling cascade, which initiates the bacterial movement towards or away from a variety of environmental signals. The “sequence-specific DNA binding transcription factor activity” directly controls gene expression, leading to physiological events that affect cellular metabolism. Indeed, 26 genes were found to encode for methyl-accepting chemotaxis proteins (MCPs) and transcriptional regulators with high expression levels *in planta* (**Table S5**). In contrast, co-downregulated DEGs were associated with essential intracellular systems. The “ATP binding” term, denoting genes involved in bioenergy substances, was most frequently represented in both BR and BX groups. Moreover, the terms “cytoplasm” and “tricarboxylic acid (TCA) cycle” were included.

Furthermore, the key functions of the *in planta* universal model were elucidated by examining the co-expression patterns shared in the studied genomes of the thee bacterial species. Consequently, 28 common DEGs were identified out of 641 genes (**Figure S3).** Similar to pairwise comparisons, over 70% of the common DEGs had identical expression tendencies, i.e., there were four co-upregulated and 18 co-downregulated genes. The functions highly required in planta in the three pathogens are listed in **Table 1**. The average relative expression (*in planta*/*in vitro* fold change) of these key genes was as follows: flagellar biosynthesis protein FlhA, 3.92-fold; C4-dicarboxylate ABC transporter, 5.94-fold; protocatechuate 3,4-dioxygenase (PCD), 3.56-fold; and 2-methylisocitrate lyase (MICL), 2.66-fold. In contrast, most co-downregulated DEGs were involved in primary metabolism (**Table S6**), such as the processing of major carbon sources and generation of high-energy bonds. Examples of such DEGs include those encoding fumarate hydratase, fructose 1,6-bisphosphatase, and enolase.

**Table 1:** List of co-upregulated DEGs in the core genome.

Description	Gene locus ID (Relative expression^a^)		
	*B. glumae*	*R. solanacearum*	*Xoo*
Flagellar biosynthesis protein FlhA	*BGLU_RS00925* (7.21-fold)	*RS_RS23845* (4.14-fold)	*XOO_RS12870* (2.00-fold)
C4-dicarboxylate ABC transporter	*BGLU_RS16715* (2.13-fold)	*RS_RS01625* (3.86-fold)	*XOO_RS05400* (25.46-fold)
Protocatechuate 3,4-dioxygenase	*BGLU_RS23755* (3.43-fold)	*RS_RS07255* (2.83-fold)	*XOO_RS02315* (4.63-fold)
2-Methylisocitrate lyase	*BGLU_RS28435* (2.25-fold)	*RS_RS17740* (2.31-fold)	*XOO_RS04315* (3.58-fold)

a Relative expression values presented as fold change *in planta/in vitro*.

### 3.3 Expression profiles of unique genomes reflect pathogen-specific mechanisms

**Figure 3:**
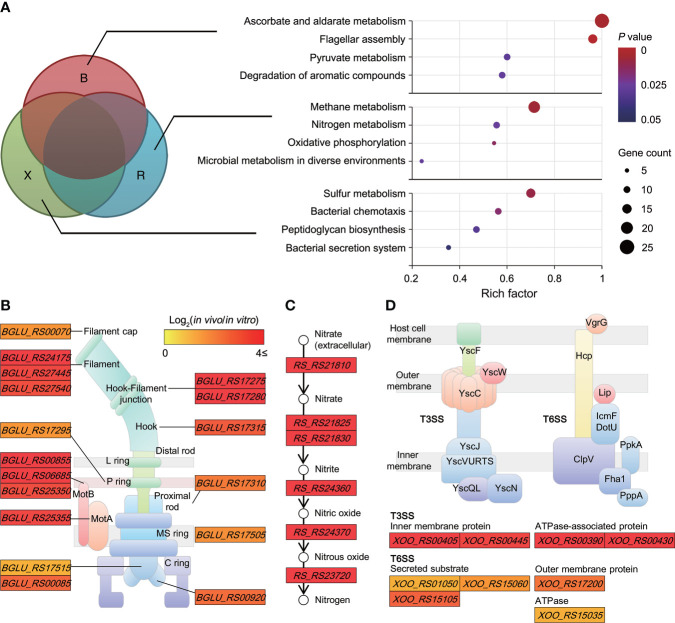
Biological pathways uniquely induced in non-orthologous groups **(A)** Scatterplot showing biological pathways significantly enriched in unique genomes (B group, **(*B*)** *glumae*; R group, *R. solanacearum*; and X group, *Xoo*). The rich factor on the *x*-axis refers to the ratio of the number of DEGs to the background number of annotated genes in the pathway. The size of the dot indicates the number of genes, while the color indicates the *P*-value range. The Cytoscape tool was used to construct networks depicting highly concentrated reactions, as follows: **(B)** flagellar assembly in **(*B*)** *glumae* BGR1; **(C)** nitrogen metabolism in *R. solanacearum* GMI1000; and **(D)** bacterial type III and VI secretion systems in *Xoo* KACC10331. Two node types are illustrated: rectangular, functional protein and circular, and biological chemical. Color differences are indicative of corresponding expression changes in log_2_(*in planta*/*in vitro*) values, with darker red representing genes upregulated *in planta*.

The biological pathways solely induced in non-orthologous groups were identified. Enrichment analysis assigned all upregulated DEGs of unique genomes to Kyoto encyclopedia of genes and genomes (KEGG) pathways (*P* ≤ 0.05). Across the non-orthologous groups, 12 non-overlapping pathways were differentially upregulated in each pathogen; the rich factors of “ascorbate and aldarate metabolism” (1.00) and “flagellar assembly” (0.96) were significantly higher than those of the other pathways (**Figure 3A**). Further, some specific functions appeared to be highly concentrated within these pathways. Most genes (25 genes) belonging to the flagellar assembly pathway were upregulated in *B. glumae*, with an average change of 8.88-fold (**Figure 3B**). The *in planta*-dependent expression of six genes upregulated in nitrate-to-nitrogen metabolism was observed in *R. solanacearum* (**Figure 3C**). Moreover, two modules of the bacterial secretion system—”type III secretion” (four genes with 14.66−109.61-fold changes) and “type VI secretion” (eight genes with 2.48−8.84-fold changes)—were specifically enriched in *Xoo* (**Figure 3D**). These pathways reflect the specific mechanisms through which each pathogen can successfully trigger plant disease.

### 3.4 The mutation of key functional genes decreases bacterial virulence

**Figure 4:**
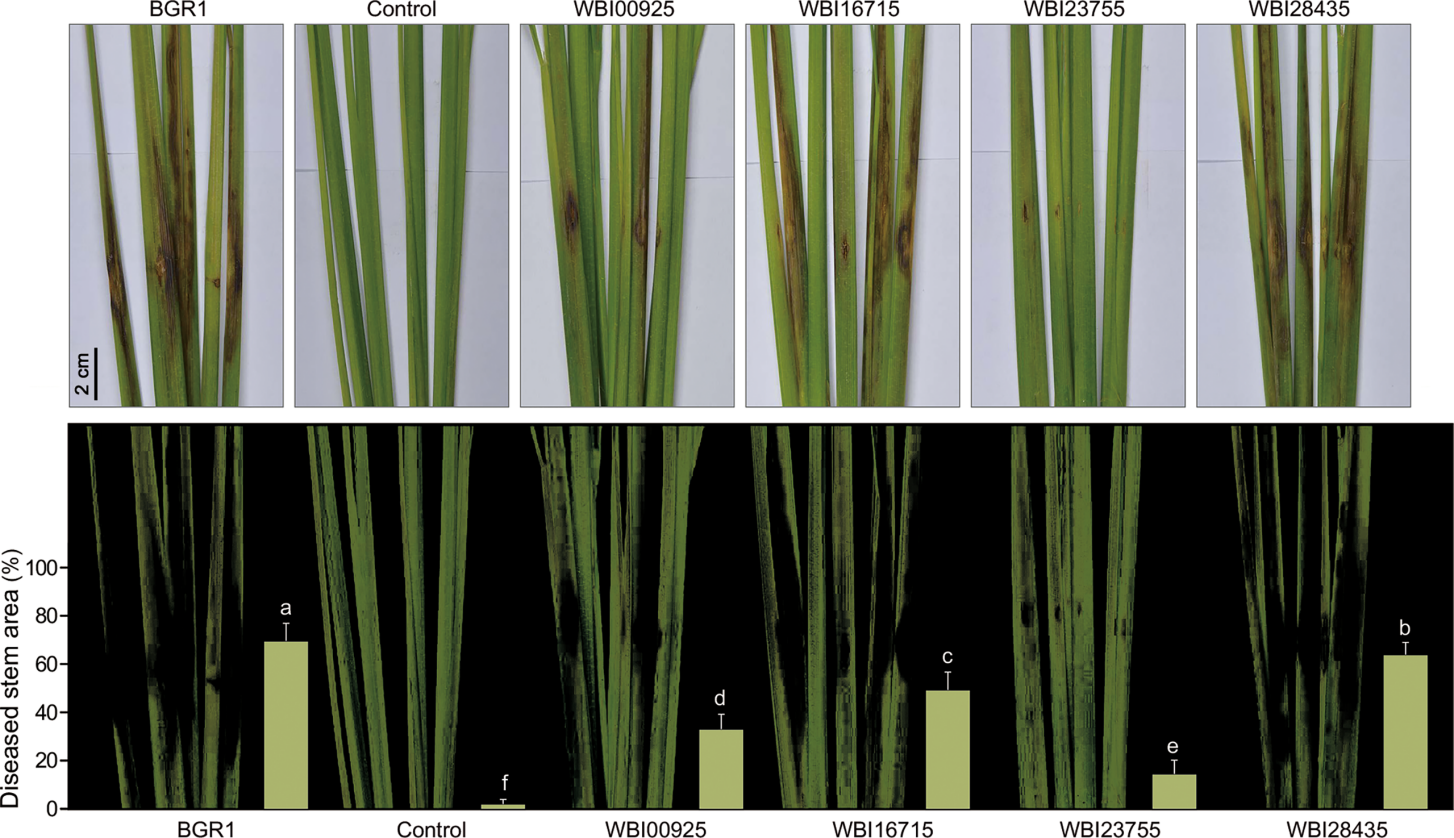
** **A single colony inoculation assay of **(B)** glumae mutants in rice stems At the vegetative stage, rice stems (cv. Dongjin) were inoculated using a syringe needle (~1 mm puncture) with bacterial colonies of the wild-type **(*B*)** *glumae* (BGR1) and the mutant strains deficient in each of the shared key functional genes (WBI00925, WBI16715, WBI23755, and WBI28435). Uninoculated plants served as negative controls. Infection courts of rice stems were photographed seven days after bacterial inoculation (upper panel). Lesion areas were captured and measured using Adobe Photoshop CS6 with the color range module (lower panel). Disease severity triggered by each strain was assessed from the percentage of diseased stem area as a bar chart. Error bars represent standard errors (*n* = 9). Different lowercase letters on the error bar indicate significant difference between treatments according to the least significant difference test at *P* ≤ 0.05.

To determine whether the co-expression patterns identified through the comparative analysis of *in planta* transcriptomes actually affected the bacterial virulence, we first constructed mutant strains of *B. glumae* (Tables S7, S8) for four key genes in the core genome (**Table 1**). During cultivation in the pure medium, growth defects of the mutant strains were not observed (**Figure S4**). We then assessed whether these mutants exhibited a reduced ability to infect rice stems using a single colony inoculation assay (**Figure 4**). *B. glumae* BGR1 infection was associated with severe disease symptoms, i.e. discoloration from rice stems to leaves. The MICL mutant WBI28435 was also virulent, triggering plant damage to an extent similar to that caused by the wild-type strain. In contrast, the C4-dicarboxylate transporter mutant WBI16715 produced only a few lesions on rice stems. The estimated disease area (%) affected by the WBI16715 mutant was approximately 20% smaller than that affected by the wild-type strain (*P* ≤ 0.05). Stems inoculated with the PCD mutant WBI23755 showed the largest healthy area, differing significantly from that of wild-type-inoculated stems (*P* ≤ 0.005). In fact, the average diseased area of WBI23755-infected plants was 14.6%, which was less than 25% of that of wild-type-infected plants. Moreover, the FlhA mutant WBI00925 induced mild disease symptoms (33.2%), with only localized <2 cm lesions surrounding the inoculation sites. The flagellar structure is an important element for bacterial virulence, since its motility enables pathogens to reach the site of infection in hosts (Kim et al., 2007). Consistently, we observed that the WBI00925 mutant did not exhibit swarming motility on 0.5% agar plates, which was similar to the quorum sensing (QS)-deficient mutant BGS2 (**Figure S5**).

The results of the virulence assay using the four *B. glumae* mutants revealed the severely impaired virulence of the WBI23755 mutant (**Figure 4**). Further, the contribution of PCD to the successful infection of plant hosts by the other pathogens was investigated. Two PCD-disrupted strains were additionally constructed in *R. solanacearum* and *Xoo* (WRI07255 and WXD02315, respectively) (**Tables S7**, **S8**). These mutants did not show any phenotypic alteration when grown on a medium compared to their respective wild-type strain.

**Figure 5:**
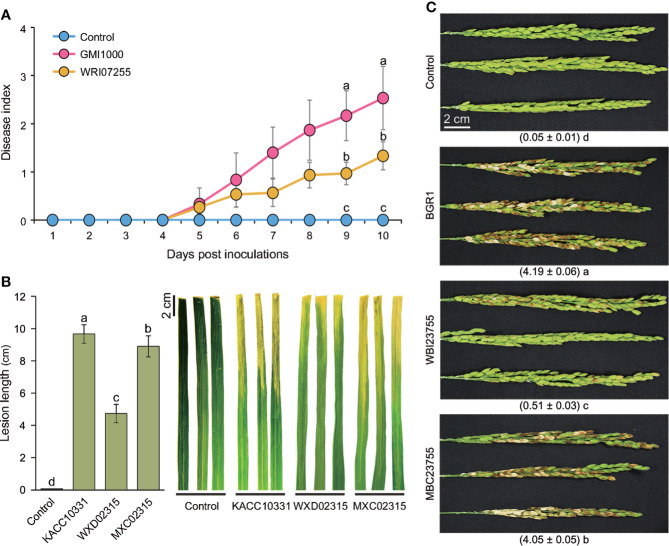
Virulence assays for PCD mutants of the three pathogens **(A)** Three- to four-week-old tomato plants were inoculated through the natural soil-soaking method with wildtype *R. solanacearum* GMI1000 and the PCD-defective mutant (WRI07255). The severity of bacterial wilt was rated daily on a disease index scale of 0 (no wilting) to 4 (76–100% wilted or dead). The points represent the average disease indices of 30 plants with standard errors. **(B)** The bar graph and error bars represent the measured average lesion lengths and the standard errors (*n* = 9) and photographs of the disease symptoms were obtained 14 days after the leaf-clipping inoculation with wild-type *Xoo* (KACC10331), the PCD defective mutant (WXD02315) and the complemented strain (MXC02315). **(C)** Bacterial suspensions of the wild-type **(*B*)** *glumae* (BGR1), the PCD-defective mutant (WBI23755) and the complemented strain (MBC23755) were inoculated into rice panicles at the flowering stage. Photographs were acquired 14 days after inoculation, and the numbers below the photographs refer to the disease index ranging from 0 (healthy panicle) to 5 (80–100% discolored panicle). In all virulence assays, an uninoculated samples served as negative controls. Significant virulence differences are marked with different lowercase letters indicating *P* ≤ 0.05 according to the least significant difference test.

Tomatoes were inoculated with *R. solanacearum* GMI1000 or WRI07255 using a natural soil-soak method. The wild-type strain completely wilted more than 70% of the inoculated tomato plants approximately 15 days after inoculation, with an average disease index of 2.9 (**Figure 5A**). Conversely, the virulence of the WRI07255 mutant was significantly reduced, resulting in an average disease index of 2.1 by the end of the assay (*P* ≤ 0.05). In *Xoo*, cultured wild-type KACC10331 and mutant WXD02315 cells were inoculated using the leaf-clipping method. The disease symptoms were recorded by measuring the length of the chlorotic to necrotic lesions 14 days after inoculation. As shown in **Figure 5B**, the WXD02315 mutant caused more attenuated disease symptoms and smaller lesions than the wild-type strain, suggesting that its ability to infect rice leaves was significantly decreased (*P* ≤ 0.05). We also investigated the disease severity of mutant WBI23755 in rice panicles where *B. glumae* BGR1 is of origin. Compared with the PCD mutants of *R. solanacearum* and *Xoo*, the WBI23755 mutant almost completely lost its virulence (**Figure 5C**). Indeed, disease severity, estimated visually by the scale score, was significantly reduced (*P* ≤ 0.05) on rice panicles inoculated with the WBI23755 mutant (disease index = 0.5), whereas the wild-type strain caused severe symptoms with a disease index of 4.2. The virulence defects in leaf-clipping assay and panicle blight assay were partially restored with the complementation strains MXC02315 and MBC23755. Thus, the biological function of PCD was found to be critical for all pathogens to completely trigger plant disease. In particular, since mutagenesis in *B. glumae* led to a significant decrease in disease damage, PCD may participate in the functional regulation of multiple virulence factors during infection.

### 3.5 PCD is involved in bacterial growth and phytotoxin biosynthesis in *B. glumae*

**Figure 6:**
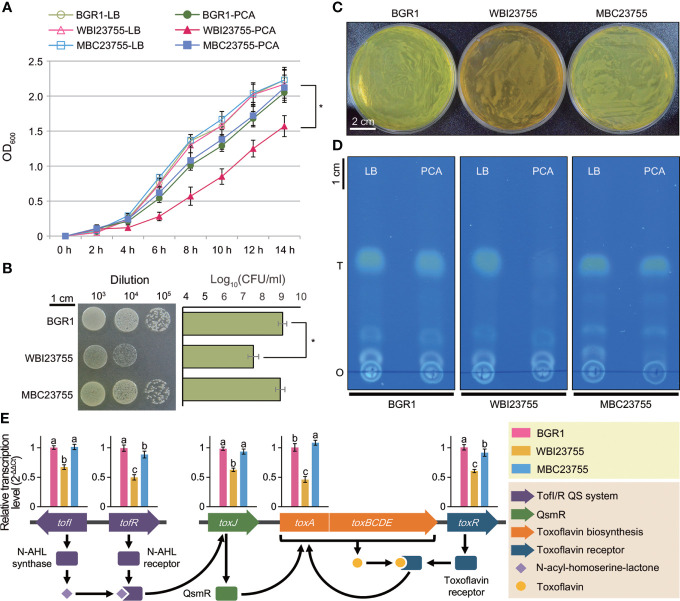
Phenotype assays of the WBI23755 mutant in the presence of PCA **(A)** The growth of each strain was monitored in LB broth with and without PCA. OD_600_ values were measured at 2 h intervals over a period of 14 h. Error bars represent the standard errors from three independent experiments. The asterisk represents a significant difference between samples (Student’s *t*-test *P* ≤ 0.05). **(B)** A spot dilution assay was conducted on LB agar plates supplemented with PCA. Bacterial cultures were serially diluted, and a 10-μl aliquot was dropped for each spot. A photograph was acquired after 24 h of incubation at 37°C. Each row and bar represents the mean of log(CFU/ml) and the standard error, respectively. **(C)** The pigmentation of the WBI23755 mutant was evaluated in the presence of PCA. Agar plates were acquired after 24 h of bacterial incubation at 37°C. The yellow pigment of BGR1 is toxoflavin. An uninoculated sample served as a negative control. **(D)** The quantitative level of toxoflavin was determined by a TLC assay. Toxoflavin produced by each strain was collected from LB broth and LB broth with PCA. A TLC plate was photographed under UV light at 365 nm (o: origin; T: toxoflavin). **(E)** The effect of PCA on the expression of major genes (*tofI*, *tofR*, *toxA*, *toxJ*, and *toxR*) of toxoflavin biosynthesis was analyzed by qPCR. The schematic diagram illustrates the distribution of genes and their regulation system for toxoflavin biosynthesis in **(*B*)** *glumae*. The relative transcription levels were calculated using the 2^−ΔΔCt^ method and are presented above each gene. Error bars represent the standard errors from three replicates. Different lowercase letters above the error bar indicate statistical significance at *P* ≤ 0.05 according to the least significant difference test. In all assays, the concentration of PCA was set to 1 mg/ml, and the MBC23755 strain was used as a positive control.

The severe virulence defect of the WBI23755 mutant prompted us to test the role of PCD in the major physiological activities under protocatechuic acid (PCA) treatment. Bacterial growth was evaluated by measuring changes in optical density (OD) and performing a spot dilution assay. There was no difference in bacterial growth between the wild-type and mutant strains in the pure medium (**Figure 6A**). However, in a PCA-containing medium (1 mg/ml), the growth rate of WBI23755 cultures after 14 h was much lower than those of wild-type cultures (OD_600_, 1.57 vs. 2.05; *P* ≤ 0.05) and MBC23755 cultures (OD_600_, 1.57 vs. 2.12; *P* ≤ 0.05). Moreover, the results of the spot dilution assay revealed an apparent decrease in WBI23755 viability during exposure to PCA in the 10^-3^ to 10^-5^ dilution range (**Figure 6B**). After incubation for 24 h, the mean viability of the WBI23755 mutant was 7.60 ± 0.27 log(colony forming unit, CFU/ml), while the mean viabilities of the wild-type and MBC23755 strains were 9.09 ± 0.22 log(CFU/ml) and 8.97 ± 0.24 log(CFU/ml), respectively.

Interestingly, the WBI23755 mutant showed a defect in toxoflavin (a phytotoxin) production on PCA-supplemented plates. Although the wild-type and MBC23755 strains produced copious amounts of toxoflavin, observed as a yellow pigment, the mutant produced a brown pigment (**Figure 6C**). The level of toxoflavin production was further evaluated using TLC. Under UV light at 365 nm, the wild-type strain displayed a strong yellow color, indicative of toxoflavin production. Conversely, in the WBI23755 mutant, abundant production of toxoflavin, similar to that in the wild-type strain, was observed in the pure medium, but it was markedly reduced in the presence of PCA (**Figure 6D**). Based on image analysis of TLC plates (**Figure S6**), the band of the WBI23755 mutant in the presence of PCA showed a significant reduction in concentration of 8.6% compared to that of the wild-type strain in pure medium. The toxoflavin production of the complementation strain (93.5% relative concentration in the presence of PCA) was similar to that of the wild-type strain. Next, quantitative real-time PCR (qPCR) was applied to measure expression changes in genes involved in the regulation and biosynthesis of toxoflavin under PCA supplementation (**Figure 6E**). The following five genes were selected: N-acyl-homoserine-lactone (AHL) autoinducer synthase, *tofI*; AHL receptor, *tofR*; QS-mediated regulator QsmR, *toxJ*; methyltransferase, *toxA*; and toxoflavin receptor, *toxR*. The relative transcription of these genes was shown to be significantly inhibited in the WBI23755 mutant compared to the wild-type strain, with fold changes ranging from 1.5–2.0 (**Figure 6E**). On the other hand, there was no significant difference in gene expression between the strains in the normal condition without PCA (**Figure S7**). The complementation strain restored the expression levels of genes under PCA supplementation. These results indicate that the interaction between PCA and PCD is directly or indirectly involved in the regulation of toxoflavin production by *B. glumae*.

## 4 Discussion

**Figure 7:**
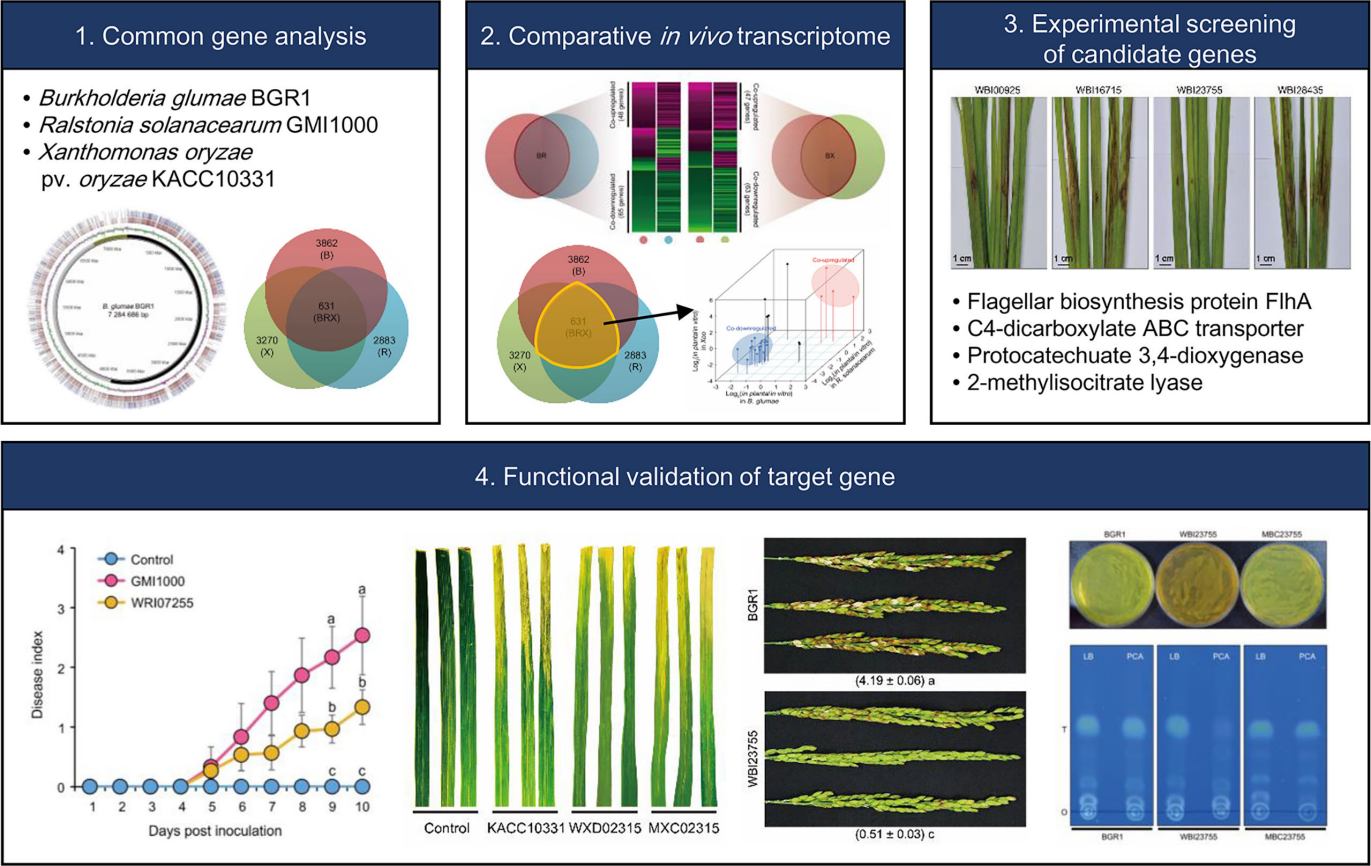
Schematic diagram representing the workflow adopted in this study to identify common cross-species virulence factor of phytopathogenic bacteria.

In recent years, the comparative analysis of transcriptomes across species has emerged as a framework for studying similarities in gene expression and could reveal novel unique and common genetic factors (Gerstein et al., 2014; Czaban et al., 2015; Sudmant et al., 2015). This concept is based on a universal model, containing a single set of organism-independent parameters that despite millions of years of evolutionary divergence, can illustrate the fundamental principles of conserved transcription (Gerstein et al., 2014; Sudmant et al., 2015). In this study, cross-species *in planta* transcriptome analysis, was used to characterize the gene expression pattern and to identify the common factors utilized by various three different phytopathogenic bacteria (*B. glumae* BGR1, *R. solanacearum* GMI1000, and *Xoo* KACC10331) to establish dynamic interactions with their hosts. The selected bacterial phytopathogen species in this study are causing serious diseases to their host and *B. glumae* shares common hosts with both *R. solanacearum* GMI1000, and *Xoo*. Therefore, the genome sequence of *B. glumae* served as cornerstone for comparisons with the other two species. Moreover, the identified common up-regulated 4 key functional genes from the *in planta* transcriptome were first tested in *B. glumae* then the influential gene locus on the virulence was verified in following mutagenesis experiments in the other two species. Ultimately the PCD was identified as a common cross-species virulence-related gene in the tested bacterial species following the workflow illustrated in **Figure 7**.

Despite the evolutionary distance of these pathogens, many genetic elements were highly conserved at a genome-wide scale (**Figure 1**). The microbial core genome supports the basic functional aspects of organisms associated with essential phenotypes (Tettelin et al., 2005). We discovered that the orthologous groups of genes included processes such as energy production and the flow of genetic information, which are crucial for bacterial life. In contrast, non-orthologous groups, dominated by proteins of unknown function and transposons, reflected the characteristics of the unique bacterial genome. Dispensable and unique genes are responsible for the overall microbial fitness in environmental niches (Tettelin et al., 2005). Indeed, the *in planta* transcriptome analysis of the unique genomes emphasized the optimized mechanisms that each pathogen developed to fully trigger plant disease (**Figure 3**). It should be noted that *B. glumae* BGR1 was originally isolated from the diseased rice grain (Lim et al., 2009). In comparison with other tissues, the rice stem provides an efficient waterway for plant pathogens to the upper plant parts (Yadeta and Thomma, 2013). *B. glumae* would attempt to reach the grain to obtain abundant nutrients by activating most of the motility-related systems. In *R. solanacearum*, nitrogen has been reported to play an important role in ATP production, detoxification, and bacterial virulence (Dalsing et al., 2015). Dalsing et al. demonstrated that nitrogen source limitation significantly decreases bacterial virulence in the tomato host (Dalsing et al., 2015). Also, the bacterial secretion system uniquely induced in *Xoo* is a major mechanism for the delivery of virulence factor to the host and environment (Furutani et al., 2009). Although the function of the type VI secretion system in *Xoo* has less been characterized, many studies recently revealed that the type VI secretion system directly affects the virulence and survival within hostile environments (Ma et al., 2014; Shyntum et al., 2015). Therefore, the *in planta*-dependent activation of non-orthologous genes as well as orthologous genes would be an essential element of convergent strategies to dominate plant hosts.

Overlaying *in planta* expression profiles onto the pan-genome revealed the co-expression patterns associated with global interactions with a hostile environment. The dominant feature of co-downregulated genes was their disproportionate involvement in energy metabolism with ATP binding (**Figure 2**). Nutrient acquisition within plant hosts poses a fundamental challenge to energy production by pathogens because of the extremely low solubility and availability of major nutrients (Jones and Wildermuth, 2011; Yadeta and Thomma, 2013). Moreover, the plant defense mechanism of nutrient allocation from infected sites contributes to the difficulty associated with the acquisition of sufficient nutrients by pathogens (Pageau et al., 2006; Schultz et al., 2013; Veillet et al., 2016). In contrast to nutrient-rich *in vitro* conditions, a lack of ATP-linked energy in hostile environments may result in the reduction of the proportion of cellular components identified in the orthologous groups, as well as in the inhibition of the associated essential functions.

The three pathogens also employed alternative strategies to overcome their survival limitations and establish pathogenicity in a hostile environment. We found that the co-expression patterns were involved in a signal transduction cascade (**Figure 2**). The ability of pathogens to dominate plant hosts depends on the functional products that mediate nutrient acquisition and resistance to defense mechanisms, as well as on the signal transduction that allows induction of the expression of these products when and where they are needed. The first step in any signal transduction is signal detection by a membrane protein. In this study, 20 MCPs, acting as membrane sensors, were found to be commonly upregulated among three pathogens. Signal recognition by MCPs provides insights into the molecular events governing the related pathways, including flagellum biosynthesis (Berleman and Bauer, 2005), degradation of xenobiotic compounds (Luu et al., 2015), and toxin production (Harkey et al., 1994). Therefore, our results revealed that the three pathogens consistently use signal transduction to respond to different hosts, infection sites, or environments. *In silico* analysis of the bacterial genome showed that 54% of 450 genomes possess signal transduction genes (Wuichet and Zhulin, 2010). The abundance of MCPs in bacteria varies with the metabolic versatility, habitat stability, and interaction with other species. Interestingly, Matilla and Krell (2018) revealed that the abundance of these genes in plant pathogens is higher than that of overall bacteria and that of animal/human pathogens. The plant condition is a complex environment with both low nutrient availability, defense mechanisms, abiotic stresses, and competition from native microorganisms. The signal transduction of plant pathogens in this hostile environment is essential to promote the colonization of more favorable niches within host tissues.

Therefore, there is a need to determine common factors among plant pathogens. Co-upregulated DEGs in the studied bacterial genomes could represent key functions within the *in planta* common virulence model (**Table 1**). The onset of bacterial motility through the production of a flagellar structure is the primary strategy for coping with environmental changes. Three pathogens showed high *flhA* expression, which encodes the export apparatus for flagellum assembly. Bacterial motility enables movement towards favorable environments, as well as harm avoidance, in response to diverse signals (Kim et al., 2007). Given nutrient limitations or considerable energy requirements, abundant carbon sources in plant hosts are likely to be attractive signals for pathogens. As central metabolites, C4-dicarboxylates, including succinate, fumarate, and malate, have been identified in various plant tissues (Janausch et al., 2002; Mellgren et al., 2009; Tamir-Ariel et al., 2011). Thus, the activation of C4-dicarboxylate transporters for the uptake of plant-derived carbon sources has been suggested as an essential strategy for the survival of pathogens in hostile environments. For example, some pathogens of the genera *Pseudomonas* and *Xanthomonas* prefer C4-dicarboxylates to other carbon sources within plants (Mellgren et al., 2009; Tamir-Ariel et al., 2011). In addition, *Pseudomonas syringae* pv. *tomato* DC3000 mutants lacking the dicarboxylate transporter do not grow to the level of the wild-type strain *in planta* (Mellgren et al., 2009). We revealed that the mutation of a C4-dicarboxylate transporter, as well as flagellar biosynthesis protein, is directly related to the virulence of *B. glumae*, indicating their importance for both survival and colonization (**Figure 4**).

In hostile environments, MICL may play another key function related to the conversion of external compounds into available resources. MICL, a signature enzyme of the methylcitrate cycle, protects microorganisms by detoxifying propionate, a cell-toxic compound abundant in soil (Lee et al., 2009). The methylcitrate cycle supplies succinate and pyruvate, which are used by pathogens to produce energy and biomass in a nutrient-limited environment (Dubey et al., 2013). Studies have revealed that MICL mutants show no significant differences in phenotype with the wild-type strain (Lee et al., 2009; Goo et al., 2017), similar to our virulence assay results (**Figure 4**). This phenomenon is due to the cross-activity of isocitrate lyase (ICL), which has evolved from an ancestral gene performing a different function by gene duplication. The mutation of MICL in *Fusarium graminearum* did not affect growth or virulence; however, the double mutation of MICL and ICL resulted in reduced virulence against plant hosts (Lee et al., 2009). Similarly, Goo et al. (2017), reported that MICL and ICL double mutants of *B. glumae* exhibited a significantly reduced oxalate production, resulting in decreased plant-mediated alkaline toxicity. Indeed, ICL of *B. glumae* (*BGLU_RS06215*) and *R. solanacearum* (*RS_RS06810*) showed high expression levels, with ≥5-fold changes *in planta* (**Table S4**). In contrast, the absence of ICL in *Xoo* led to the highest *in planta* expression of MICL among three pathogens for an alternative requirement (**Table 1**). Although the role of MICL has been studied less extensively in pathogenic bacteria than in fungi, the 50% sequence identity between fungal and bacterial MICL genes suggests the importance of further studying this gene as a virulence factor.

The other gene of interest encodes PCD, an enzyme responsible for PCA ring cleavage. Defects in PCD resulted in a significant reduction in virulence at the major infection site in all three pathogens (**Figure 5**). PCA is regarded as a beneficial phenolic compound because of its antibacterial activity (Chao and Yin, 2009); it is widely distributed across edible plants and human foods (Kakkar and Bais, 2014). Here, we demonstrated an intimate relationship between PCD and bacterial resistance to PCA by measuring the effect of PCA on bacterial growth (**Figures 6A**,**B**). The expression of PCD, a key enzyme in the β-ketoadipate pathway, may represent an essential countermeasure of pathogens against plant defense mechanisms. Thus, plant hosts synthesize PCA to eliminate invaders from their tissues (Kakkar and Bais, 2014), whereas pathogens may express PCD to protect themselves from antibacterial activity. Although the β-ketoadipate pathway is present in most bacteria that recognize aromatic compounds, it is mainly distributed among soil microorganisms (Harwood and Parales, 1996; Li et al., 2010). Further, a BLASTp-based survey (>30% identity, >30% coverage, and <1.0 × 10^-5^ E value cutoffs to define a match) of the distribution of PCD across 30 representative plant pathogens deposited in the NCBI genome database highlighted the ubiquity of this gene in most pathogens (22 matches, 73.3%) (**Table S9**). At the genus level, plant pathogens were grouped into the same clade, respectively (**Figure S8**).

Interestingly, we confirmed that the production of toxoflavin by the WBI23755 mutant was reduced in the presence of PCA (**Figure 6D**). Toxoflavin causes chlorotic damage to the panicles and inhibits the growth of leaves and roots of rice, leading to severe crop losses (Jeong et al., 2003). The gene cluster of the *tox* operon and the genetic regulation for toxoflavin biosynthesis and transport have been well-characterized in *B. glumae.* The major regulators, a LysR-type, ToxR and ToxJ regulators, were shown to be involved in the activation of *tox* operons for biosynthesis and transport. The process is also regulated by the quorum sensing system through *tofI*, and *tofR*, *via N*-acyl homoserine lactone signals (Mannaa et al., 2018). In this study we have shown that PCD defects resulted in down-regulation of the *tofI*, *tofR, toxJ, toxA* and *toxR* which could explain the reduction of toxoflavin production. Nevertheless, the relationship between PCD and toxoflavin production remains unclear. This enzyme may be involved in the metabolism of pigments and toxoflavin in *B. glumae*. The qPCR results revealed a negative effect of PCA on the expression of major genes involved in toxoflavin production, depending on the presence of PCD (**Figure 6E**). We are currently investigating the nature of the brown pigment produced by the WBI23755 mutant under PCA supplementation (**Figure 6C**). Karki et al. (2012), reported that plant pathogenic *B. glumae* 411gr-6 produces various pigments (brown, purple, and fluorescent compounds) different from toxoflavin under specific conditions. It has been suggested that these alternative pigments are likely to be involved in resistance to environmental stresses (Karki et al., 2012).

Although the exact roles of PCD in relation to phytopathogenic bacterial virulence is yet unknown, the fact that it is involved in the degradation of aromatic compounds supports the suggestion that it could be employing bacteria with ecological advantage. Previous studies have also shown that another important phytopathogen, the *Agrobacterium tumefaciens* could utilize the same function to promote its pathogenicity allowing the bacteria to overcome the toxicity of the aromatic compounds present around wounded plant tissues that might be detrimental to bacterial survival (Parke, 1997; Parke, 2000). However, further work is required to unravel the exact link between PCD and the bacterial phytopathogen as our investigation is suggesting its ubiquity as a virulence factor.

## 5 Conclusion

Our study attempted to construct a universal virulence model for representative plant pathogenic bacteria, namely *B. glumae*, *R. solanacearum*, and *Xoo*. Comparative analysis of *in planta* transcriptomes among species allowed us to identify putative common factors related to global plant-pathogen interactions, which could not be found by independent analysis of each pathogen. Co-expression patterns showed consistent activation of MCP-based signal transduction to recognize the external conditions within hosts. The following key functions in the core genome showed alternative strategies for the survival and colonization of hostile environments: (i) bacterial movement to preferred niches; (ii) uptake of carbon sources to overcome nutrient limitation; (iii) conversion of external compounds into available resources; and (iv) degradation of plant-derived antibacterial substances. Among these, the disruption of PCD, a novel virulence factor, led to mild disease symptoms in plants infected with all pathogens, and in particular, it had a critical effect on toxoflavin production by *B. glumae*. In conclusion, comparative *in planta* transcriptome analysis across plant pathogens can be a useful approach for understanding the pathogenic lifestyle within plant hosts. As such, it can contribute to unraveling key virulence factors, thereby aiding in the development of novel plant defense strategies.

## Data availability statement

The datasets presented in this study can be found in online repositories. The names of the repository/repositories and accession number(s) can be found in the article/**Supplementary Material**.

## Author contributions

JP and Y-SS conceived and designed the project. JP, SL, NK, and GH conducted the experiments. D-SP, Se-WL, and Sa-WL helped conduct some of the experiments. JP, MM, HJ, and H-HL analyzed the data. JP, MM, and Y-SS wrote the manuscript with contributions from other authors. All authors read and approved the manuscript.

## Funding

This research was supported by the Basic Science Research Program of the National Research Foundation (NRF), funded by the Ministry of Education (2022R1A2C1003190), (2020R1A6A1A03047729**),** Republic of Korea and by the National Institute of Fisheries Science (NIFS), funded by the Ministry of Oceans and Fisheries of the South Korea (R2022045).

## Conflict of interest

The authors declare that the research was conducted in the absence of any commercial or financial relationships that could be construed as a potential conflict of interest.

## Publisher’s note

All claims expressed in this article are solely those of the authors and do not necessarily represent those of their affiliated organizations, or those of the publisher, the editors and the reviewers. Any product that may be evaluated in this article, or claim that may be made by its manufacturer, is not guaranteed or endorsed by the publisher.
