# Supplementary material for: Genome-guided comparative in planta transcriptome analyses for identifying cross-species common virulence factors in bacterial phytopathogens
Source: Front Plant Sci. 2022 Nov 16;13:1030720. doi: 10.3389/fpls.2022.1030720 (PMC9709210; doi:10.3389/fpls.2022.1030720)
Supplement: Supplementary file 1 [file DataSheet_1.docx]

Supplementary Material

**Data source for bioinformatics**

Genome sequences and annotation information were downloaded from the NCBI genome server (ftp://ftp.ncbi.nlm.nih.gov/genomes/) as follows: *Burkholderia glumae* BGR1, GCF_000022645.2 (Lim et al., 2009); *Ralstonia solanacearum* GMI1000, GCF_000009125.1 (Salanoubat et al., 2002); *Xanthomonas oryzae* pv. *oryzae* (*Xoo*) KACC10331, GCF_000007385.1 (Lee et al., 2005). For comparative *in planta* transcriptome analyses, two expression profiling datasets were downloaded from the GEO database (http://www.ncbi.nlm.nih.gov/geo/): RNA-seq library of *B. glumae* BGR1, GSM832378 (Kim et al., 2014); and microarray library of *R. solanacearum* GMI1000, GSE33657 (Jacobs et al., 2012). The *B. glumae* RNA-seq dataset included three independent replicates of *in planta* and *in vitro* libraries (Kim et al., 2014). From the *R. solanacearum* dataset, eight libraries of rich-medium and *in planta* conditions (full-blown disease in tomato) at 28°C were used (Jacobs et al., 2012).

**RNA-seq of *Xoo* KACC10331**

*Xoo* KACC10331 was incubated in peptone sucrose (PS) medium at 30°C for two days. The final concentration of the subculture was adjusted to an optical density (OD) of 0.5 at 600 nm. Cultured cells were washed twice with sterile water and resuspended in distilled water. Vegetative-stage leaves of rice (cv. Dongjin) were inoculated with the *Xoo* KACC10331 suspension using the leaf-clipping method. The inoculated plants were grown in a greenhouse (30°C during the day and 25°C during the night) for 10 days. The infected rice leaves were segmented in a cold room (4°C) into strips of approximately 3 cm in length and then centrifuged at 2000 × *g* and 4°C for 5 min in 15 ml of conical tubes (SPL Lifesciences, Pocheon, Korea) to collect *in planta* bacterial pellets. The pellets contained cells at a concentration of 1.0 × 10^9^ to 1.5 × 10^9^ CFU/ml and were primarily composed of *Xoo* KACC10331 cells. For *in vitro* growth, *Xoo* KACC10331 cultures in the PS medium were adjusted to an OD_600_ of 1.0–1.5, corresponding to a concentration of 1.0 × 10^9^ to 1.5 × 10^9^ CFU/ml. The pellets were resuspended in RNAprotect™ bacteria reagent (Qiagen, Valencia, CA, USA). The experiment was conducted using three independent replicates.

Next, total RNA was extracted from *in planta* and *in vitro* pellets using an RNeasy midi kit (Qiagen), according to the manufacturer’s protocols. Residual genomic DNA was removed using an RNase-free DNase set (Qiagen). The MICROBExpress™ bacterial mRNA enrichment kit (Ambion, Austin, TX, USA) was used to remove bacterial ribosomal RNA from the total RNA samples. Libraries for RNA-seq were prepared using the Illumina TruSeq™ RNA sample prep kit (Illumina, San Diego, CA, USA) with a standard low-throughput protocol. RNA-seq was performed using the Illumina HiSeq2000 instrument at the National Instrumentation Center for Environmental Management (NICEM, Seoul, Korea).

**Kyoto encyclopedia of genes and genomes (KEGG) pathway enrichment analysis**

KEGG is a useful database for evaluating high-throughput sequencing results in terms of activated/inactivated biological pathways (http://www.genome.jp/kegg/). Pathway information was used to identify the biological systems induced in the non-orthologous groups of pathogens. Incomplete pathways forming a partial network (≤5 genes) were excluded to remove false positives. Each non-overlapping KEGG pathway was matched to *in planta*-dependent differentially expressed genes (DEGs) in the unique genome using an in-house developed python script. A hypergeometric distribution, obtained with the phyper function, was used to perform enrichment analysis for comparison with the reference gene background (Qureshi and Sacan, 2013). The formula applied for this analysis is shown below.

$$P=1-\sum_{i=0}^{m-1} \frac{\binom{m}{i}\binom{N-M}{n-i}}{\binom{N}{n}}$$

where *N* represents the number of KEGG-annotated genes in the whole genome, and *n* represents the number of target genes in *N*. *M* represents the number of single pathway-annotated genes, and *m* represents the number of target genes in *M*. KEGG pathways with *P* ≤ 0.05 were considered to be significantly enriched.

**Bacterial strains, plasmids, and growth conditions**

The bacterial strains and plasmid constructs used in this study are listed in Table S7. *B. glumae* and *Escherichia coli* strains were cultivated in Luria-Bertani (LB) broth at 37°C. The *R. solanacearum* and *Xoo* strains were cultivated at 28°C in casamino acid peptone glucose and PS medium, respectively. Yeast extract dextrose CaCO_3_ (YDC) medium was used for triparental mating between *R. solanacearum* and *E. coli* HB101. All liquid cultures were incubated in a shaker-incubator at 200 rpm. When required, the media were supplemented with antibiotics as follows: ampicillin (Amp), 100 μg/ml; apramycin (Apm), 100 μg/ml; cephalexin (Cp), 20 μg/ml; kanamycin (Km), 50 or 100 μg/ml; rifampin (Rif), 50 or 100 μg/ml; and tetracycline (Tc), 15 μg/ml.

**Construction of mutant strains**

Standard techniques were used for DNA manipulation, cloning, restriction digestion, and agarose gel electrophoresis (Sambrook and Russell, 2001). Details on the primers used for the mutagenesis experiments are provided in Table S8.

The internal regions of co-upregulated DEGs were amplified from *B. glumae* BGR1 and inserted into the pGEM-T Easy vector (Promega, Fitchburg, WI, USA). After sequencing to confirm the correctness of ligation, the amplified fragments were digested with *Eco*RI and *Kpn*I (New England Biolabs, Ipswich, MA, USA) and inserted into the pVIK112 suicide vector (Kalogeraki and Winans, 1997). *E. coli* DH5α λ*pir* competent cells were transformed with the recombinant pVIK112 plasmids and then cultured in a Km-supplemented (50 μg/ml) LB medium. The amplified plasmids were introduced into *E. coli* S17-1 λ*pir* before transferring to *B. glumae* BGR1 using the biparental mating method. Finally, the identity of *B. glumae* mutants that were able to grow on a medium supplemented with two antibiotics (Km, 100 μg/ml and Rif, 100 μg/ml) was confirmed by PCR annealing to an internal sequence (Lacfuse) of the pVIK112 backbone and sequences upstream of the genes.

In *R. solanacearum*, the mutagenesis of protocatechuate 3,4-dioxygenase (PCD) was carried out according to previously described methods (Lee et al., 2001). Briefly, two fragments of the *RS_RS07255* gene spanning upstream and downstream were separately amplified from *R. solanacearum* GMI1000. The nucleotide sequences between the upstream and the downstream fragments were complementary to the DNA sequences of the Km resistance gene cassette. One such cassette was amplified from the pMKm2 plasmid. The three amplified DNA fragments were mixed at an equimolar ratio and overlap extension PCR (OE-PCR) was performed to fuse the three PCR products. The fused product was cloned into pGEM-T Easy vector and subsequently subcloned into the broad-host-range pRK415 vector (Keen et al., 1988) to generate pRIRS07255. The latter was introduced into the wild-type strain by triparental mating in YDC medium, with pRK2013 acting as a helper vector. Mutants sensitive to Tc (15 μg/ml) but resistant to Km (50 μg/ml) were selected.

To construct a PCD mutant in *Xoo* according to Cai et al. (2017), the upstream and downstream regions of *XOO_RS02315* were amplified and fused by OE-PCR. The correct PCR products were ligated into the suicide vector pK18*mobsacB* (Schäfer et al., 1994) using *Eco*RI and *Hind*III (New England Biolabs) digestion. Using the biparental mating method, the recombinant pXDRS02315 plasmid was introduced into the wild-type strain to generate single-crossover mutants on Km- and Cp-supplemented medium (50 μg/ml and 20 μg/ml, respectively). Next, the single-crossover mutants were incubated in a PS medium for two days before growing on a medium containing Cp (20 μg/ml) and 10% sucrose (w/v) to select double-crossover mutants. The correctness of the PCD-disrupted mutants was confirmed by PCR.

**Complementation of PCD mutant strains in *B. glumae* and *Xoo***

The DNA fragments containing the *BGLU_RS23755* gene (705 bp) of *B. glumae* and the *XOO_RS02315* gene (729 bp) of *Xoo* were PCR amplified from the genomic DNA, using the primer sets BCRS23755_H/BCRS23755_B and XCRS02315_E/XCRS02315_B, respectively (Table S8). After sequence verification, these PCR products were excised and ligated into pRK415 and pBBR1MCS2 vectors (Keen et al., 1988; Luo and Farrand, 1999), resulting in the cloned genes being oriented in the same direction as the *lac* promoter of two vectors (Table S7). The complementation plasmids, pBCRS23755 and pXCRS02315, were introduced into the mutant WBI23755 and WXD02315, respectively, via the conjugation of *E. coli* S17-1. The appropriate transformation of the complementation plasmid was confirmed by a colony PCR technique.

**Swarming motility assay**

The swarming motility assay was performed on LB agar (0.5% agar, w/v). The wild-type *B. glumae* BGR1, FlhA mutant WBI00925, and quorum sensing (QS)-deficient mutant BGS2 were incubated at 37°C overnight in 3 ml of LB liquid medium. Thereafter, 1 ml of stationary phase culture was centrifuged at 1000 × *g* for 2 min and washed twice with fresh LB broth. The bacterial pellets were resuspended in 100 μl of LB broth. Each cell suspension (5 μl) was spotted on swarming assay plates. After 24 h of incubation at 37°C, the bacterial movement was assessed.

**TLC assay**

The level of toxoflavin production by *B. glumae* strains under protocatechuic acid (PCA) supplementation was determined by a TLC assay. Cultured cells (1 ml) were centrifuged at 12,000 × *g* for 1 min; the supernatant (500 μl) was transferred into a new tube containing 500 μl of chloroform. After vortexing on a Vortex-Genie 2 (VWR, Chicago, IL, USA), the mixture was again centrifuged at 12,000 × *g* for 10 min. Next, 500 μl of the infranatant was transferred to a new tube and dried in a centrifugal vaporizer (Tokyo Rikakikai, Tokyo, Japan). The solutes were dissolved in 10 μl of 80% methanol, and an aliquot of each solution was repetitively applied onto a TLC plate (Merck Millipore, Darmstadt, Germany). The TLC plates were placed in a chamber (solvent, 95:5 chloroform: methanol) for 20 min. 3UV™ multi-wavelength lamps (UVP, Upland, CA, USA) were used to examine the band patterns. To compare fluorescence intensity of band patterns, image analysis of TLC plates was conducted using the quantitative TLC (qTLC) tool following the detailed tutorial presented in Mac Fhionnlaoich et al. (2018). Assuming that the toxoflavin concentration of the wild-type strain in LB broth was 100%, the relative concentration of the band in each sample was quantified.

**Quantitative real-time PCR (qPCR) analysis**

PCA (1 mg/ml) was added to 1:100 dilutions of overnight cultures of *B. glumae* strains; the mixtures were then incubated at 37°C for 2 h. Total RNA was extracted using the RNeasy midi kit (Qiagen) and treated with RNase-free DNase (Qiagen). DNase-treated total RNA (2–2.5 μg) was used as starting material to generate cDNA with the SuperScript™ III first-strand kit (Invitrogen, Carlsbad, CA, USA). The generated cDNA was diluted to a concentration of 15–20 ng/μl in DEPC-treated water. The sequences of primers used for qPCR are listed in Table S8. Each reaction mixture was prepared as follows: TOPreal™ qPCR 2X premix (Enzynomics, Seoul, Korea), 10 μl; cDNA, 1 μl; primers, 2 μl each; and distilled water, 7 μl. qPCR analysis was performed using a Rotor-gene Q instrument (Qiagen) with the following protocol: 10 min at 95°C, followed by 50 cycles of PCR (95°C for 10 s, 60°C for 15 s, and 72°C for 20 s). The relative transcription levels were determined using the 2^-ΔΔCt^ method with the 16S rRNA gene for normalization.**SUPPLEMENTARY FIGURES**


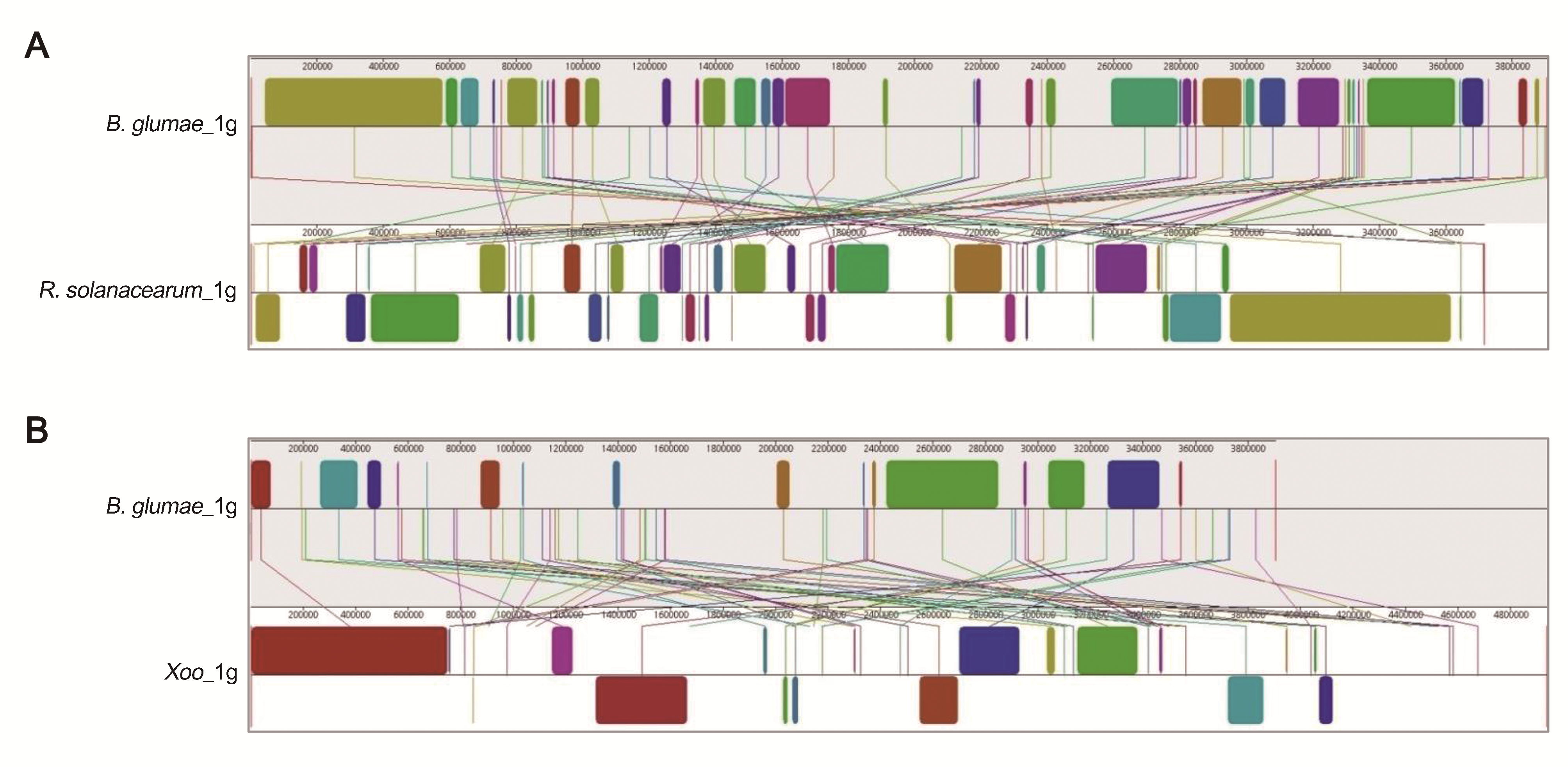


**Figure S1. Genome rearrangements among chromosome 1 replicons of the three pathogens**

The chromosome 1 replicon of *B. glumae* BGR1 was used as a reference. A Mauve was used to align the reference sequence to the chromosome replicons of **(A)** *R. solanacearum* GMI1000 and **(B)** *Xoo* KACC10331. Colored blocks represent similarity profiles within the genome sequence, denoted as locally collinear blocks (LCBs). The highly homologous LCBs between pathogens are labeled with identical colors. LCBs below the central line of each pathogen indicate regions in the reverse complement orientation.


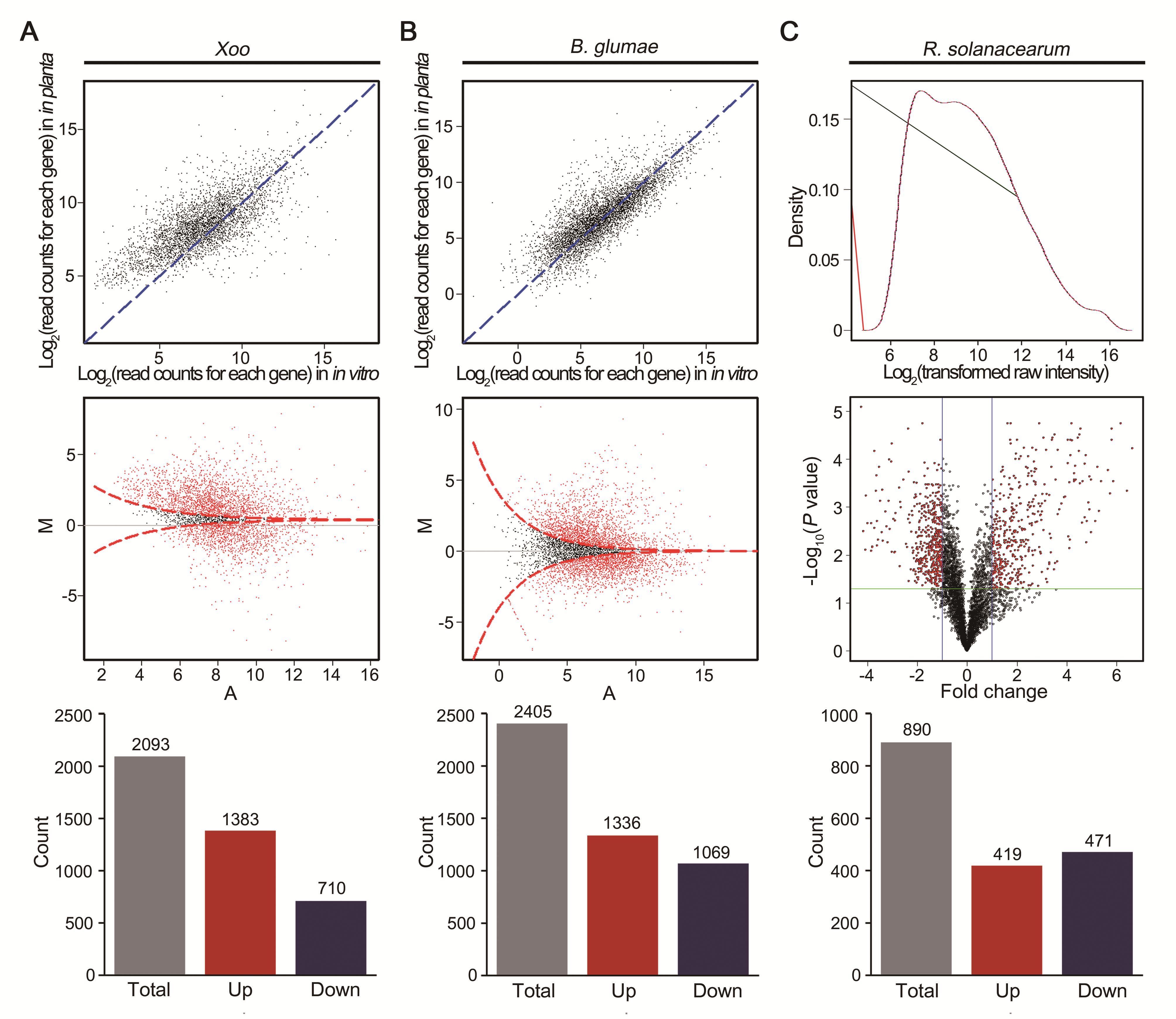


**Figure S2. Overview of DEGs of *in planta* transcriptomes**

The distribution of expressed genes in **(A)** *Xoo* and **(B)** *B. glumae* is represented in a scatterplot and MA-plot. The scatterplot depicts the comparison of gene expression levels, expressed as normalized read counts, between *in vitro* (*x*-axis) and *in planta* (*y*-axis) conditions. Red points in the MA-plot reflect significant differences between conditions with a false discovery rate (FDR) < 0.05 as determined by the MARS method. **(C)** In *R. solanacearum*, a density plot illustrates the distribution of gene expression levels in the color channels of each array. The expression intensity of genes is plotted on the *x*-axis and the density values on the *y*-axis. The log_2_(*in planta*/*in vitro*) and their corresponding –log(*P*-value) of all genes were taken for the construction of a volcano plot. Black dots denote genes that have not changed significantly. The horizontal green line and vertical blue line depict the cut-off values for the fold changes and *P*-value, respectively. In all three pathogens, the same criteria of an FDR < 0.05 and log_2_(*in planta*/*in vitro*) ≥ 1 were used for DEG identification. Bar charts at the bottom row illustrate the distribution of DEGs. Red and blue bars denote the number of upregulated and downregulated genes, respectively.


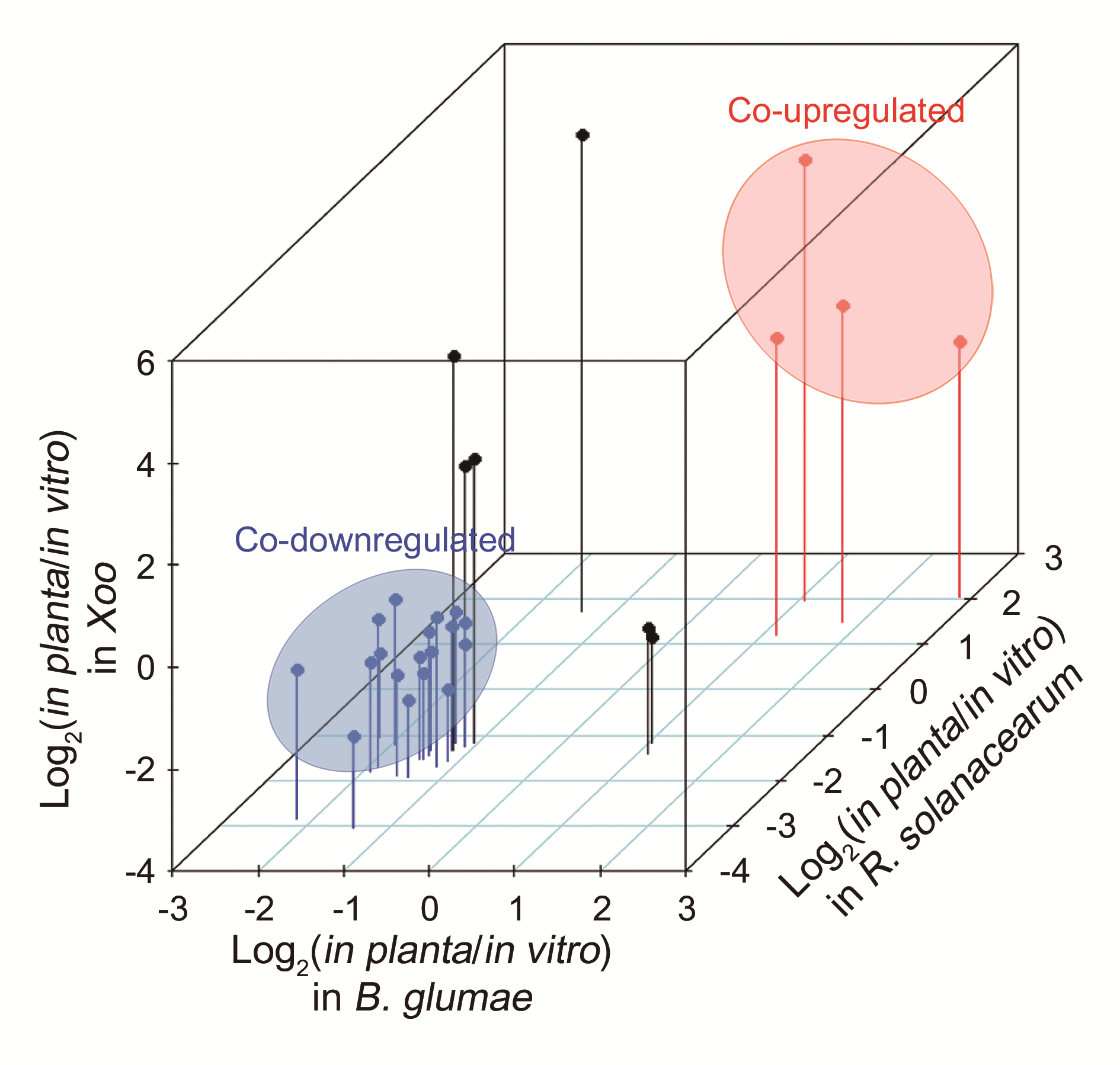


**Figure S3. Co-expression patterns of the core genome across the three pathogens**

Each node represents a common DEG determined by *in planta* transcriptome analysis of all three pathogens. The *x*-, *y*-, and *z*-axes define the relative expression, presented as log_2_(*in planta*/*in vitro*). DEGs that followed identical expression tendencies were grouped into colored circles. Red and blue nodes indicate co-upregulated and co-downregulated DEGs *in planta*, respectively.


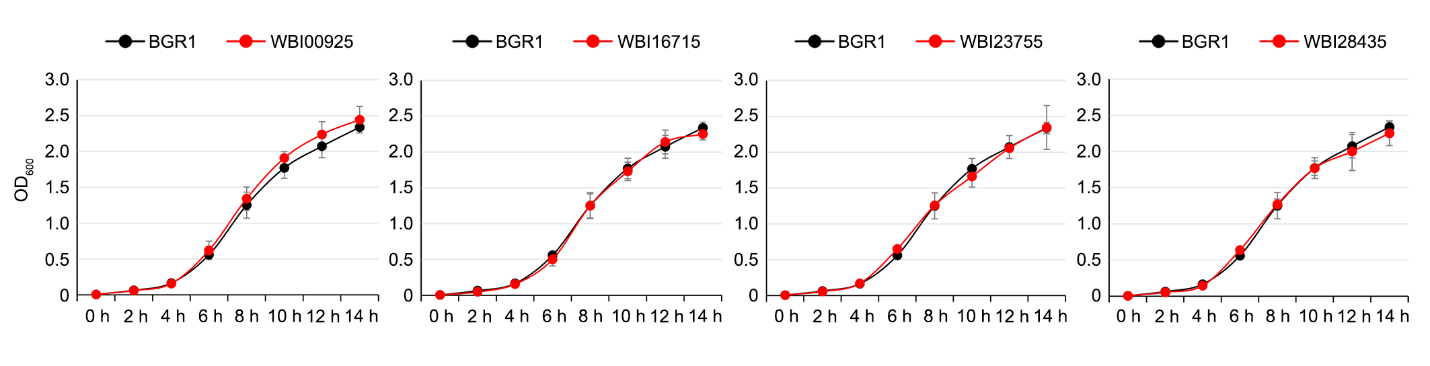


**Figure S4. Growth curve of the *B. glumae* BGR1 and mutant strains**

The growth of the wild-type *B. glumae* BGR1 and four mutants (WBI00925, WBI16715, WBI23755, and WBI28435) was monitored in pure LB broth. OD_600_ values were measured at 2 h intervals over a period of 14 h. Error bars represent the standard errors from three independent experiments.


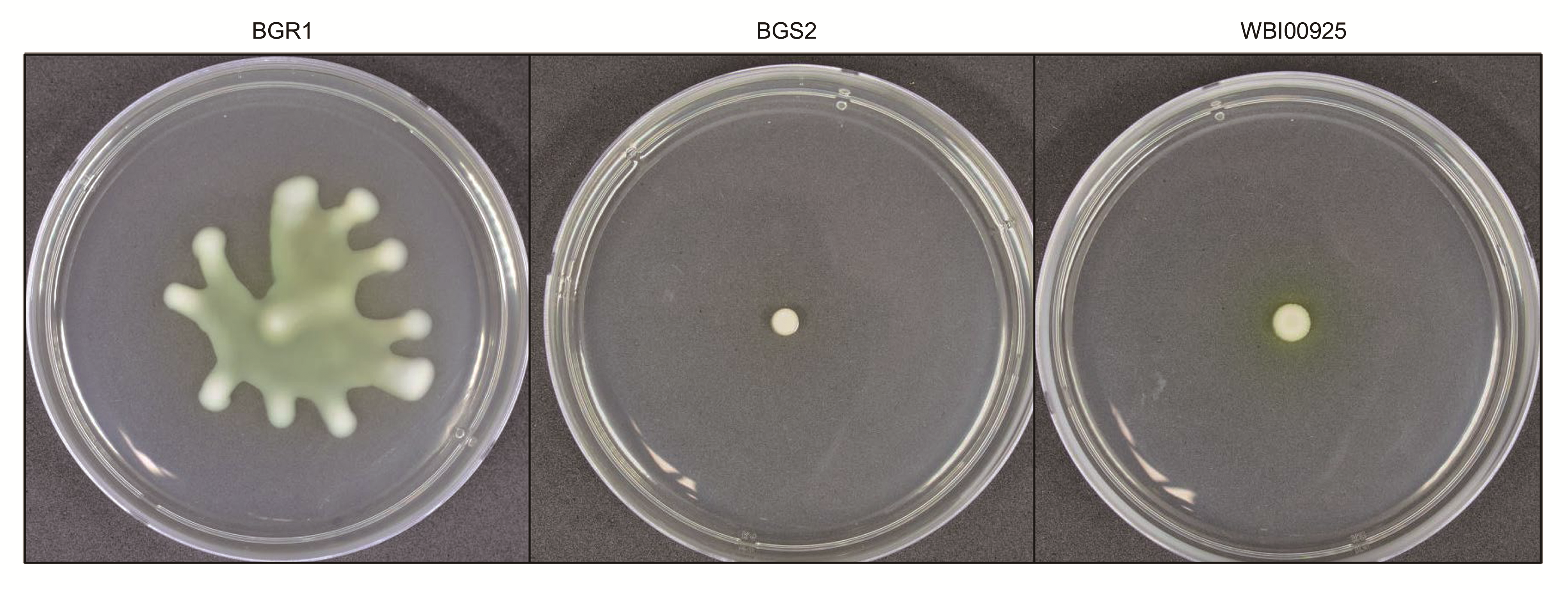


**Figure S5. Swarming motility of the flagellar biosynthesis protein FlhA-disrupted mutant**

A swarming motility assay was performed to inspect the bacterial motility of the WBI00925 mutant. Cultured bacterial cells were seeded onto 0.5% agar plates, and swarming motility was measured after 24 h of incubation at 37°C. The uncovered plates were photographed by irradiating a standardized light source D65 (Daylight 6500K) in a light box (Super Light-VI, BoTeck, Korea). *B. glumae* BGR1 and QS-deficient mutant BGS2 were used as the positive and negative control, respectively.


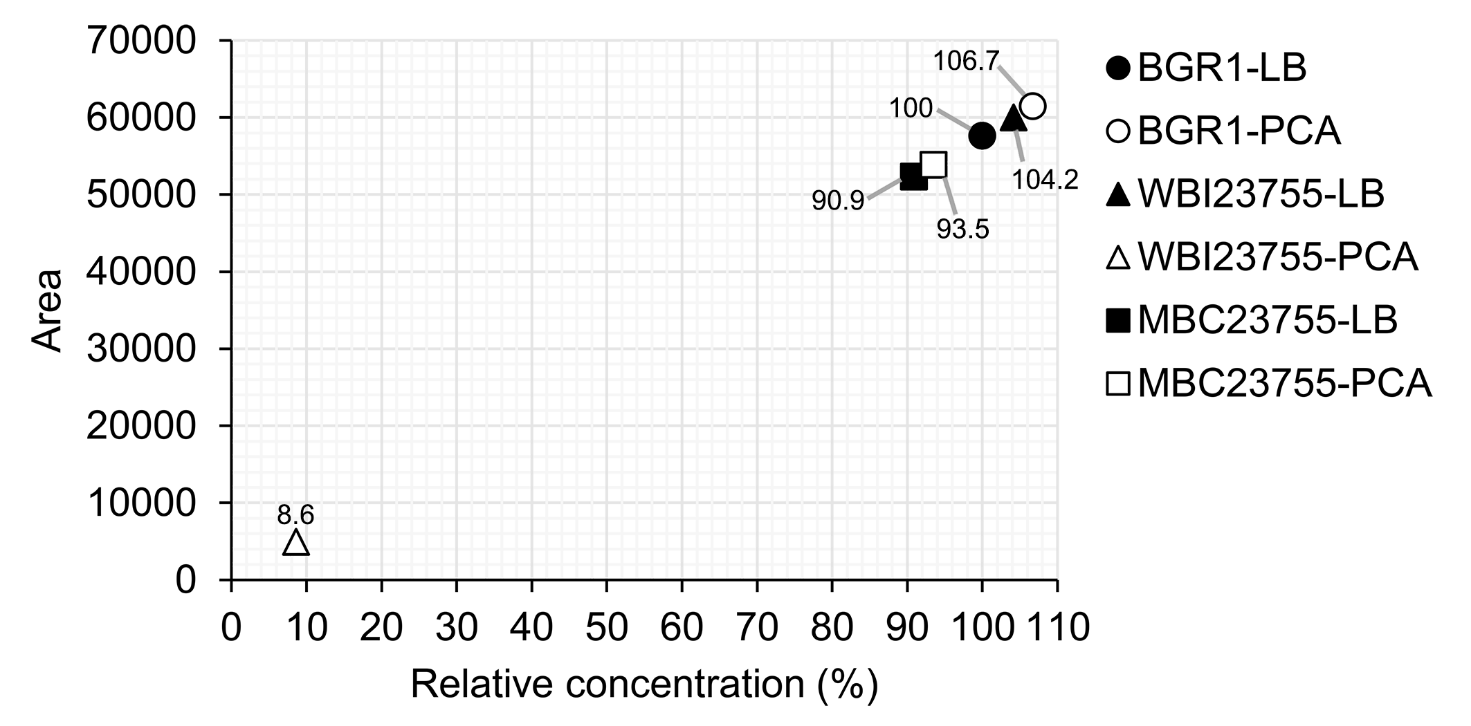


**Figure S6. TLC image-based quantification of toxoflavin**

Toxoflavin quantification assay was performed using the qTLC tool. The *x*-axis represents the relative toxoflavin concentration of samples compared to that of BGR1-LB sample as control TLC band (100%). The *y*-axis represents the pixel area of each TLC band, with higher numbers indicating abundant production of toxoflavin. The relative concentration values are indicated at spots.


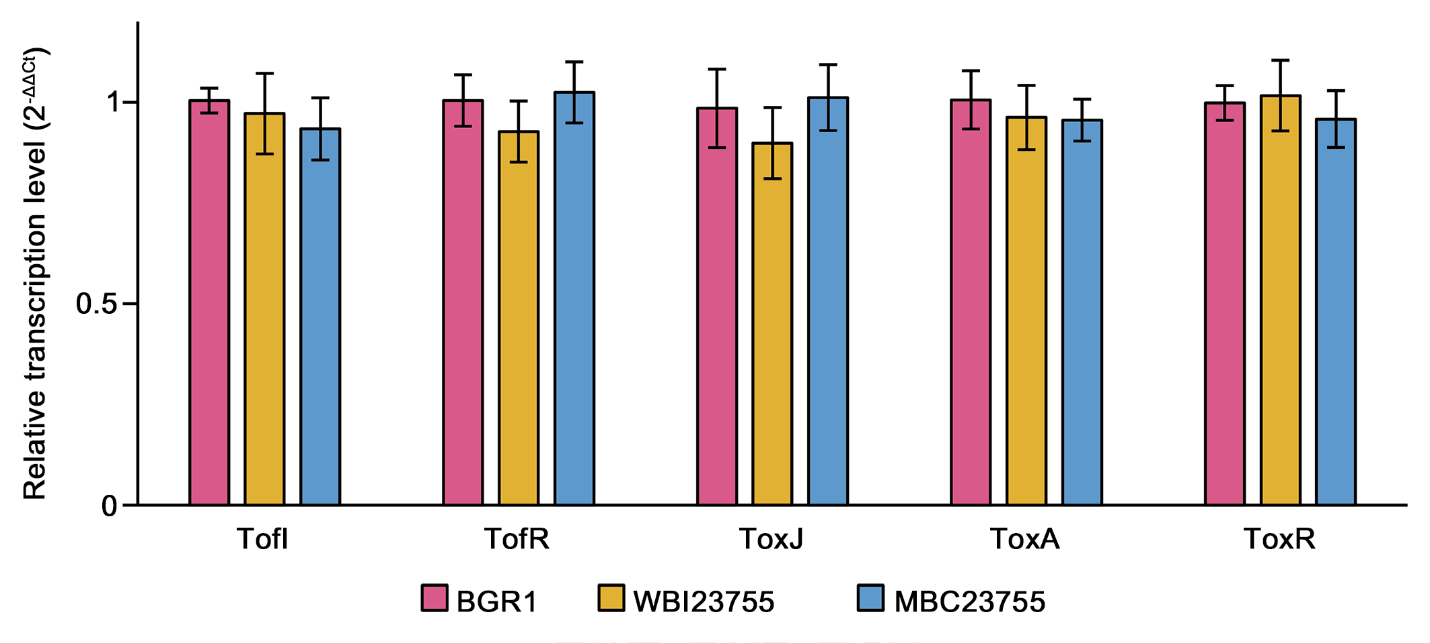


**Figure S7. Relative transcription level of genes related to toxoflavin biosynthesis in LB medium.**

Under the normal condition without PCA, the expression of toxoflavin biosynthesis genes (*tofI*, *tofR*, *toxJ*, *toxA*, and *toxR*) in *B. glumae* BGR1, WBI23755, and MBC23755 strains was determined by qPCR. The relative transcription levels were calculated using the 2^−ΔΔCt^ method. Error bars represent the standard errors from three replicates. There were no statistical significances between treatments at *P* <0.05 according to ANOVA.


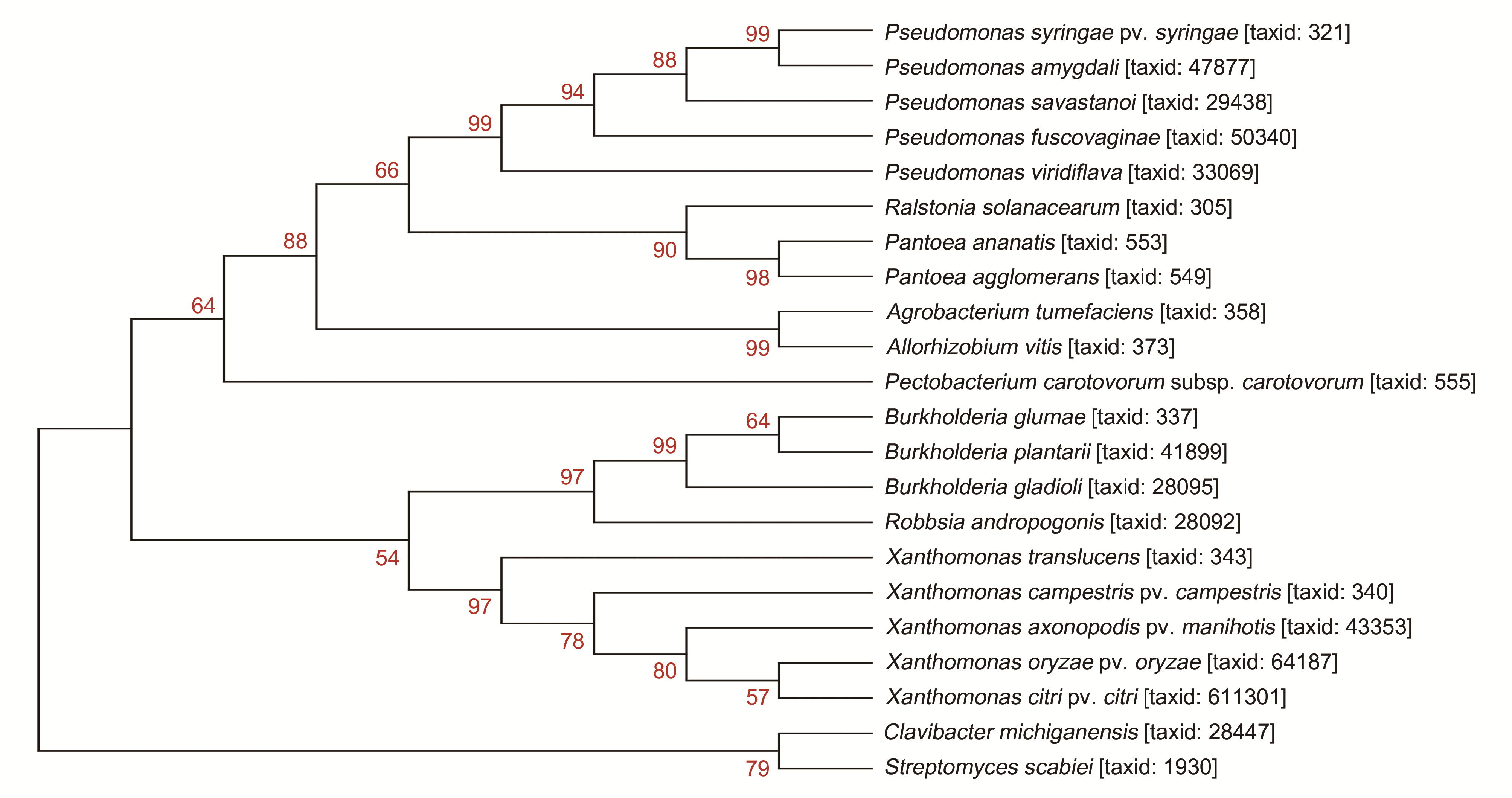


**Figure S8. Phylogenetic tree based on PCD amino acid sequences**

The amino acid sequences of PCD genes from 22 plant pathogens were used for phylogenetic analysis as follows: *Agrobacterium*, 1 pathogen; *Allorhizobium*, 1 pathogen; *Burkholderia*, 3 pathogens; *Clavibacter*, 1 pathogen; *Pantoea*, 2 pathogens; *Pectobacterium*, 1 pathogen; *Pseudomonas*, 5 pathogens; *Ralstonia*, 1 pathogen; *Robbsia*, 1 pathogen; *Streptomyces*, 1 pathogen; and *Xanthomonas*, 5 pathogens. The percentage of iterations clustered together in the bootstrap test based on 1000 replications are shown next to the corresponding branch and values greater than 50% are denoted. The NCBI taxonomy ID of each pathogen is shown in parentheses.**SUPPLEMENTARY TABLES**

**Table S1. Summary of genome statistics of the three plant pathogens**

| Organism | Replicon | Size (Mb) | GC (%) | Gene | rRNA | Protein | RefSeq^a^ |
| --- | --- | --- | --- | --- | --- | --- | --- |
| *B. glumae* BGR1 | Chromosome 1 | 3.91 | 68.1 | 3568 | 9 | 3429 | NC_012724.2 |
|  | Chromosome 2 | 2.83 | 68.8 | 2198 | 6 | 2131 | NC_012721.2 |
|  | Plasmid 1 | 0.13 | 60.6 | 129 | 0 | 117 | NC_012723.1 |
|  | Plasmid 2 | 0.14 | 63.2 | 112 | 0 | 106 | NC_012718.1 |
|  | Plasmid 3 | 0.14 | 62.7 | 114 | 0 | 104 | NC_012720.2 |
|  | Plasmid 4 | 0.13 | 62.7 | 113 | 0 | 108 | NC_012725.2 |
| *R. solanacearum* GMI1000 | Chromosome 1 | 3.72 | 67.0 | 3429 | 9 | 3316 | NC_003295.1 |
|  | Plasmid 1 | 2.09 | 66.9 | 1583 | 3 | 1533 | NC_003296.1 |
| *Xoo* KACC10331 | Chromosome 1 | 4.94 | 63.7 | 4568 | 6 | 4055 | NC_006834.1 |

^a^Reference sequence ID in the GenBank database.

**Table S2. The top five COG categories in the orthologous and non-orthologous groups**

| COG category | Description | Average percentage (%)^a^ | |
| --- | --- | --- | --- |
|  |  | Orthologous group | Non-orthologous group |
| J | Translation, ribosomal structure and biogenesis | 11.38 | 2.15 |
| C | Energy production and conversion | 8.66 | 4.26 |
| H | Coenzyme transport and metabolism | 6.19 | 3.40 |
| F | Nucleotide transport and metabolism | 3.73 | 1.04 |
| L | Replication, recombination and repair | 4.49 | 2.61 |
| M | Cell wall/membrane/envelope biogenesis | 4.41 | 6.32 |
| K | Transcription | 5.02 | 8.29 |
| R | General function prediction only | 5.91 | 9.41 |
| S | Function unknown | 1.84 | 5.93 |
| X | Mobilome: prophages, transposons | 2.03 | 9.86 |

^a^Average percentage in the particular functional category of groups relative to all genes of 25 COG categories.

**Table S3. Detailed information on RNA-seq libraries of *Xoo* KACC10331**

| Sample | Orientation^a^ | Length | Quality^b^ | Raw reads | Filtered reads^c^ | Mapped reads in genes |
| --- | --- | --- | --- | --- | --- | --- |
| *In vitro* 1 | R1 | 126 | 36 | 29,295,580 | 24,998,325 | 32,017,127 |
|  | R2 | 126 | 36 | 29,295,580 | 24,998,325 |  |
| *In vitro* 2 | R1 | 126 | 36 | 22,671,210 | 20,918,453 | 25,268,913 |
|  | R2 | 126 | 36 | 22,671,210 | 20,918,453 |  |
| *In vitro* 3 | R1 | 126 | 36 | 19,208,178 | 18,488,368 | 22,281,081 |
|  | R2 | 126 | 36 | 19,208,178 | 18,488,368 |  |
| *In planta* 1 | R1 | 126 | 36 | 24,937,253 | 22,977,498 | 14,757,938 |
|  | R2 | 126 | 36 | 24,937,253 | 22,977,498 |  |
| *In planta* 2 | R1 | 126 | 36 | 21,466,176 | 19,785,231 | 12,215,998 |
|  | R2 | 126 | 36 | 21,466,176 | 19,785,231 |  |
| *In planta* 3 | R1 | 126 | 36 | 17,833,318 | 16,295,579 | 10,534,935 |
|  | R2 | 126 | 36 | 17,833,318 | 16,295,579 |  |

^a^R1, forward strand reads; R2, reverse strand reads.

^b^An average Phred score.

^c^Reads filtered to a minimum Phred score of 28 in >50% lengths.**Table S4. List of common DEGs with identical expression patterns**

| Orthologous group | Gene set 1 | | Gene set 2 | | Expression tendency^b^ |
| --- | --- | --- | --- | --- | --- |
|  | Gene locus ID | Log_2_(*in planta*/*in vitro*)^a^ | Gene locus ID | Log_2_(*in planta* /*in vitro*) |  |
| BR group | *BGLU_RS00280* | 1.67 | RS_RS16745 | 1.70 | Up |
|  | *BGLU_RS00285* | 1.53 | RS_RS16740 | 1.13 | Up |
|  | *BGLU_RS00355* | -2.20 | RS_RS16620 | -1.06 | Down |
|  | *BGLU_RS00385* | -2.24 | RS_RS16540 | -1.91 | Down |
|  | *BGLU_RS00390* | -3.00 | *RS_RS16535* | -1.09 | Down |
|  | *BGLU_RS00435* | 5.79 | *RS_RS15715* | 1.38 | Up |
|  | *BGLU_RS00470* | -2.19 | *RS_RS16510* | -2.85 | Down |
|  | *BGLU_RS00475* | -1.75 | *RS_RS16505* | -1.76 | Down |
|  | *BGLU_RS00480* | -1.44 | *RS_RS16500* | -3.02 | Down |
|  | *BGLU_RS00850* | 3.12 | *RS_RS23935* | 1.33 | Up |
|  | *BGLU_RS00925* | 2.85 | *RS_RS23845* | 2.05 | Up |
|  | *BGLU_RS01925* | -1.18 | *RS_RS20650* | -1.82 | Down |
|  | *BGLU_RS01930* | -1.17 | *RS_RS20655* | -1.65 | Down |
|  | *BGLU_RS01940* | -2.11 | *RS_RS20665* | -2.02 | Down |
|  | *BGLU_RS01945* | -1.73 | *RS_RS20670* | -1.36 | Down |
|  | *BGLU_RS01950* | -1.66 | *RS_RS20675* | -1.32 | Down |
|  | *BGLU_RS01960* | -1.55 | *RS_RS20685* | -2.07 | Down |
|  | *BGLU_RS01965* | -1.88 | *RS_RS20690* | -1.69 | Down |
|  | *BGLU_RS01975* | -1.13 | *RS_RS20700* | -1.58 | Down |
|  | *BGLU_RS01980* | -1.97 | *RS_RS20750* | -2.01 | Down |
|  | *BGLU_RS01985* | -1.20 | *RS_RS20760* | -1.70 | Down |
|  | *BGLU_RS01990* | -1.44 | *RS_RS20765* | -1.63 | Down |
|  | *BGLU_RS02000* | -1.14 | *RS_RS20785* | -1.20 | Down |
|  | *BGLU_RS02045* | 1.39 | *RS_RS17670* | 1.49 | Up |
|  | *BGLU_RS02520* | -1.05 | *RS_RS13960* | -1.53 | Down |
|  | *BGLU_RS02535* | -1.02 | *RS_RS13945* | -1.26 | Down |
|  | *BGLU_RS02540* | -1.50 | *RS_RS13940* | -1.52 | Down |
|  | *BGLU_RS02545* | -2.28 | *RS_RS13935* | -1.76 | Down |
|  | *BGLU_RS02655* | -1.46 | *RS_RS13845* | -1.19 | Down |
|  | *BGLU_RS02685* | -1.20 | *RS_RS13760* | -1.35 | Down |
|  | *BGLU_RS02700* | 2.16 | *RS_RS16410* | 1.41 | Up |
|  | *BGLU_RS02810* | -1.20 | *RS_RS02390* | -1.37 | Down |
|  | *BGLU_RS03025* | -1.17 | *RS_RS13585* | -2.04 | Down |
|  | *BGLU_RS03165* | -1.46 | *RS_RS13200* | -1.09 | Down |
|  | *BGLU_RS03350* | -1.65 | *RS_RS03705* | -1.28 | Down |
|  | *BGLU_RS03430* | -1.37 | *RS_RS03795* | -1.06 | Down |
|  | *BGLU_RS03820* | -2.48 | *RS_RS03500* | -2.00 | Down |
|  | *BGLU_RS03825* | -1.64 | *RS_RS03505* | -1.51 | Down |
|  | *BGLU_RS04000* | -1.55 | *RS_RS24615* | -2.03 | Down |
|  | *BGLU_RS04195* | -1.47 | *RS_RS10665* | -1.33 | Down |
|  | *BGLU_RS04230* | -1.23 | *RS_RS10525* | -1.17 | Down |
|  | *BGLU_RS05405* | 2.27 | *RS_RS24095* | 2.16 | Up |
|  | *BGLU_RS06215* | 4.26 | *RS_RS06810* | 2.46 | Up |
|  | *BGLU_RS06220* | 1.89 | *RS_RS04735* | 1.62 | Up |
|  | *BGLU_RS06500* | -1.40 | *RS_RS24615* | -2.03 | Down |
|  | *BGLU_RS06690* | 6.52 | *RS_RS23935* | 1.33 | Up |
|  | *BGLU_RS06785* | -1.27 | *RS_RS08685* | -1.11 | Down |
|  | *BGLU_RS06835* | -2.29 | *RS_RS08815* | -1.11 | Down |
|  | *BGLU_RS06955* | 1.46 | *RS_RS16450* | 2.37 | Up |
|  | *BGLU_RS06960* | 1.27 | *RS_RS16445* | 3.52 | Up |
|  | *BGLU_RS07080* | -1.12 | *RS_RS06655* | -1.16 | Down |
|  | *BGLU_RS08460* | 3.46 | *RS_RS06545* | 1.13 | Up |
|  | *BGLU_RS08850* | 1.12 | *RS_RS24095* | 2.16 | Up |
|  | *BGLU_RS09310* | -1.15 | *RS_RS20060* | -1.02 | Down |
|  | *BGLU_RS09385* | 1.43 | *RS_RS06750* | 1.65 | Up |
|  | *BGLU_RS09940* | 1.04 | *RS_RS13990* | 2.07 | Up |
|  | *BGLU_RS09955* | -1.41 | *RS_RS06370* | -1.91 | Down |
|  | *BGLU_RS09960* | -1.44 | *RS_RS06365* | -1.52 | Down |
|  | *BGLU_RS09965* | -1.55 | *RS_RS06360* | -1.90 | Down |
|  | *BGLU_RS11190* | 3.18 | *RS_RS06215* | 1.53 | Up |
|  | *BGLU_RS11195* | 2.25 | *RS_RS06210* | 1.48 | Up |
|  | *BGLU_RS11200* | 1.99 | *RS_RS06205* | 2.41 | Up |
|  | *BGLU_RS11205* | 1.53 | *RS_RS06200* | 1.95 | Up |
|  | *BGLU_RS12840* | -1.10 | *RS_RS05735* | -1.26 | Down |
|  | *BGLU_RS12875* | -1.07 | *RS_RS05695* | -1.64 | Down |
|  | *BGLU_RS12930* | -1.22 | *RS_RS05620* | -1.06 | Down |
|  | *BGLU_RS13365* | -1.66 | *RS_RS20130* | -1.63 | Down |
|  | *BGLU_RS13400* | -1.42 | *RS_RS09830* | -1.46 | Down |
|  | *BGLU_RS13560* | -1.36 | *RS_RS10100* | -1.09 | Down |
|  | *BGLU_RS14135* | -1.91 | *RS_RS04900* | -1.81 | Down |
|  | *BGLU_RS14185* | -1.75 | *RS_RS04840* | -1.16 | Down |
|  | *BGLU_RS14695* | -1.04 | *RS_RS11840* | -1.50 | Down |
|  | *BGLU_RS14880* | -1.02 | *RS_RS12120* | -1.14 | Down |
|  | *BGLU_RS15510* | -1.61 | *RS_RS17530* | -1.38 | Down |
|  | *BGLU_RS15590* | -1.25 | *RS_RS02880* | -1.19 | Down |
|  | *BGLU_RS15720* | 1.19 | *RS_RS02740* | 2.07 | Up |
|  | *BGLU_RS15835* | 3.26 | *RS_RS07425* | 1.72 | Up |
|  | *BGLU_RS15915* | -1.05 | *RS_RS15295* | -2.37 | Down |
|  | *BGLU_RS15930* | -1.62 | *RS_RS15300* | -2.90 | Down |
|  | *BGLU_RS15935* | -1.17 | *RS_RS15335* | -1.76 | Down |
|  | *BGLU_RS16140* | -1.06 | *RS_RS02270* | -1.17 | Down |
|  | *BGLU_RS16715* | 1.09 | *RS_RS01625* | 1.95 | Up |
|  | *BGLU_RS16865* | -1.96 | *RS_RS01480* | -1.20 | Down |
|  | *BGLU_RS17170* | -1.21 | *RS_RS12800* | -1.13 | Down |
|  | *BGLU_RS17510* | 2.34 | *RS_RS19040* | 1.34 | Up |
|  | *BGLU_RS18040* | 2.52 | *RS_RS05360* | 4.08 | Up |
|  | *BGLU_RS18045* | 3.05 | *RS_RS21070* | 5.15 | Up |
|  | *BGLU_RS18655* | -1.20 | *RS_RS20675* | -1.32 | Down |
|  | *BGLU_RS18795* | 3.25 | *RS_RS24890* | 1.03 | Up |
|  | *BGLU_RS18900* | -2.02 | *RS_RS03125* | -2.17 | Down |
|  | *BGLU_RS19485* | 1.43 | *RS_RS07365* | 1.33 | Up |
|  | *BGLU_RS19720* | 1.30 | *RS_RS21080* | 2.03 | Up |
|  | *BGLU_RS19725* | 1.72 | *RS_RS21075* | 1.64 | Up |
|  | *BGLU_RS19735* | 4.59 | *RS_RS21070* | 5.15 | Up |
|  | *BGLU_RS20280* | 1.55 | *RS_RS03840* | 2.46 | Up |
|  | *BGLU_RS20335* | 1.72 | *RS_RS20360* | 1.94 | Up |
|  | *BGLU_RS20775* | 2.89 | *RS_RS17455* | 1.41 | Up |
|  | *BGLU_RS20955* | 2.08 | *RS_RS01005* | 5.87 | Up |
|  | *BGLU_RS21575* | 1.57 | *RS_RS00960* | 2.34 | Up |
|  | *BGLU_RS23160* | -1.90 | *RS_RS20130* | -1.63 | Down |
|  | *BGLU_RS23755* | 1.78 | *RS_RS07255* | 1.50 | Up |
|  | *BGLU_RS23845* | 2.63 | *RS_RS21190* | 1.83 | Up |
|  | *BGLU_RS23955* | 1.58 | *RS_RS00950* | 2.51 | Up |
|  | *BGLU_RS24295* | 1.17 | *RS_RS22230* | 2.70 | Up |
|  | *BGLU_RS24895* | 1.84 | *RS_RS18220* | 1.60 | Up |
|  | *BGLU_RS25385* | 2.71 | *RS_RS18305* | 2.18 | Up |
|  | *BGLU_RS26860* | 1.70 | *RS_RS24860* | 1.14 | Up |
|  | *BGLU_RS27745* | 4.86 | *RS_RS00950* | 2.51 | Up |
|  | *BGLU_RS27820* | 1.41 | *RS_RS07565* | 2.67 | Up |
|  | *BGLU_RS28180* | 4.34 | *RS_RS19975* | 1.17 | Up |
|  | *BGLU_RS28435* | 1.17 | *RS_RS17740* | 1.21 | Up |
|  | *BGLU_RS31170* | -1.12 | *RS_RS16115* | -1.25 | Down |
|  | *BGLU_RS31185* | -2.27 | *RS_RS16115* | -1.25 | Down |
| BX group | *BGLU_RS00040* | -1.10 | *XOO_RS02120* | -1.98 | Down |
|  | *BGLU_RS00330* | -2.04 | *XOO_RS03525* | -1.08 | Down |
|  | *BGLU_RS00345* | -1.48 | *XOO_RS03540* | -2.16 | Down |
|  | *BGLU_RS00350* | -1.41 | *XOO_RS03545* | -1.87 | Down |
|  | *BGLU_RS00355* | -2.20 | *XOO_RS03550* | -2.35 | Down |
|  | *BGLU_RS00470* | -2.19 | *XOO_RS17560* | -1.07 | Down |
|  | *BGLU_RS00480* | -1.44 | *XOO_RS08810* | -2.22 | Down |
|  | *BGLU_RS00810* | 1.02 | *XOO_RS20415* | 1.03 | Up |
|  | *BGLU_RS00875* | 2.45 | *XOO_RS14045* | 2.26 | Up |
|  | *BGLU_RS00925* | 2.85 | *XOO_RS12870* | 1.00 | Up |
|  | *BGLU_RS01475* | 1.16 | *XOO_RS11770* | 2.34 | Up |
|  | *BGLU_RS01965* | -1.88 | *XOO_RS17170* | -1.09 | Down |
|  | *BGLU_RS01975* | -1.13 | *XOO_RS17160* | -2.60 | Down |
|  | *BGLU_RS01985* | -1.20 | *XOO_RS17385* | -1.06 | Down |
|  | *BGLU_RS02350* | -1.34 | *XOO_RS18895* | -1.09 | Down |
|  | *BGLU_RS02540* | -1.50 | *XOO_RS02685* | -2.00 | Down |
|  | *BGLU_RS02685* | -1.20 | *XOO_RS05685* | -1.57 | Down |
|  | *BGLU_RS02690* | -2.46 | *XOO_RS05820* | -1.67 | Down |
|  | *BGLU_RS02740* | -1.11 | *XOO_RS16440* | -1.24 | Down |
|  | *BGLU_RS03240* | -1.73 | *XOO_RS03860* | -4.13 | Down |
|  | *BGLU_RS03565* | -4.30 | *XOO_RS21210* | -2.20 | Down |
|  | *BGLU_RS03570* | -4.31 | *XOO_RS21205* | -2.28 | Down |
|  | *BGLU_RS03970* | -1.57 | *XOO_RS19075* | -1.40 | Down |
|  | *BGLU_RS03975* | -1.33 | *XOO_RS05960* | -1.24 | Down |
|  | *BGLU_RS04095* | 1.01 | *XOO_RS13725* | 1.51 | Up |
|  | *BGLU_RS04195* | -1.47 | *XOO_RS00080* | -2.05 | Down |
|  | *BGLU_RS04410* | -1.07 | *XOO_RS10085* | -2.17 | Down |
|  | *BGLU_RS04465* | -1.04 | *XOO_RS03835* | -1.54 | Down |
|  | *BGLU_RS04890* | -1.93 | *XOO_RS03710* | -1.27 | Down |
|  | *BGLU_RS04975* | 1.98 | *XOO_RS14715* | 1.80 | Up |
|  | *BGLU_RS05265* | 5.52 | *XOO_RS09835* | 2.01 | Up |
|  | *BGLU_RS06715* | 2.37 | *XOO_RS02070* | 3.84 | Up |
|  | *BGLU_RS07030* | -1.24 | *XOO_RS14425* | -2.24 | Down |
|  | *BGLU_RS07195* | -2.79 | *XOO_RS05160* | -1.90 | Down |
|  | *BGLU_RS08735* | 2.38 | *XOO_RS08315* | 3.62 | Up |
|  | *BGLU_RS09955* | -1.41 | *XOO_RS09955* | -2.50 | Down |
|  | *BGLU_RS09960* | -1.44 | *XOO_RS09960* | -2.30 | Down |
|  | *BGLU_RS09965* | -1.55 | *XOO_RS09965* | -2.02 | Down |
|  | *BGLU_RS11100* | 1.69 | *XOO_RS14045* | 2.26 | Up |
|  | *BGLU_RS11120* | -1.27 | *XOO_RS04015* | -1.39 | Down |
|  | *BGLU_RS11395* | 3.40 | *XOO_RS14045* | 2.26 | Up |
|  | *BGLU_RS11420* | 1.95 | *XOO_RS14035* | 1.46 | Up |
|  | *BGLU_RS11475* | 1.81 | *XOO_RS11255* | 3.89 | Up |
|  | *BGLU_RS12035* | 1.77 | *XOO_RS21430* | 1.87 | Up |
|  | *BGLU_RS12840* | -1.10 | *XOO_RS08010* | -1.57 | Down |
|  | *BGLU_RS12930* | -1.22 | *XOO_RS14640* | -2.15 | Down |
|  | *BGLU_RS12985* | -1.13 | *XOO_RS14175* | -2.16 | Down |
|  | *BGLU_RS13275* | 1.05 | *XOO_RS11815* | 2.88 | Up |
|  | *BGLU_RS13400* | -1.42 | *XOO_RS11505* | -1.56 | Down |
|  | *BGLU_RS13485* | -1.19 | *XOO_RS18700* | -1.81 | Down |
|  | *BGLU_RS13635* | -1.64 | *XOO_RS15950* | -1.50 | Down |
|  | *BGLU_RS13640* | -1.17 | *XOO_RS15955* | -1.61 | Down |
|  | *BGLU_RS13645* | -1.42 | *XOO_RS15960* | -1.77 | Down |
|  | *BGLU_RS13655* | -1.62 | *XOO_RS15970* | -1.71 | Down |
|  | *BGLU_RS13660* | -1.05 | *XOO_RS15975* | -1.36 | Down |
|  | *BGLU_RS14135* | -1.91 | *XOO_RS12340* | -1.86 | Down |
|  | *BGLU_RS14660* | -2.00 | *XOO_RS04580* | -1.70 | Down |
|  | *BGLU_RS14730* | -1.16 | *XOO_RS16215* | -1.23 | Down |
|  | *BGLU_RS15505* | -1.85 | *XOO_RS19135* | -1.77 | Down |
|  | *BGLU_RS15590* | -1.25 | *XOO_RS15885* | -1.42 | Down |
|  | *BGLU_RS15695* | -1.04 | *XOO_RS07720* | -2.46 | Down |
|  | *BGLU_RS16635* | -1.26 | *XOO_RS07120* | -3.06 | Down |
|  | *BGLU_RS16715* | 1.09 | *XOO_RS05400* | 4.67 | Up |
|  | *BGLU_RS16865* | -1.96 | *XOO_RS03740* | -1.14 | Down |
|  | *BGLU_RS17095* | -1.73 | *XOO_RS10120* | -1.51 | Down |
|  | *BGLU_RS17870* | 1.46 | *XOO_RS13405* | 1.28 | Up |
|  | *BGLU_RS17995* | -2.10 | *XOO_RS18280* | -1.46 | Down |
|  | *BGLU_RS18000* | -2.08 | *XOO_RS18250* | -1.64 | Down |
|  | *BGLU_RS18480* | 1.97 | *XOO_RS14045* | 2.26 | Up |
|  | *BGLU_RS18510* | 1.59 | *XOO_RS14045* | 2.26 | Up |
|  | *BGLU_RS18605* | -3.34 | *XOO_RS00110* | -1.14 | Down |
|  | *BGLU_RS18935* | 1.92 | *XOO_RS14045* | 2.26 | Up |
|  | *BGLU_RS19030* | 1.78 | *XOO_RS00380* | 4.72 | Up |
|  | *BGLU_RS19050* | 1.20 | *XOO_RS00400* | 5.80 | Up |
|  | *BGLU_RS20095* | 4.26 | *XOO_RS14035* | 1.46 | Up |
|  | *BGLU_RS21895* | -1.04 | *XOO_RS04650* | -1.25 | Down |
|  | *BGLU_RS21930* | -2.27 | *XOO_RS05590* | -2.47 | Down |
|  | *BGLU_RS22035* | -1.46 | *XOO_RS18210* | -1.53 | Down |
|  | *BGLU_RS22280* | 2.60 | *XOO_RS14035* | 1.46 | Up |
|  | *BGLU_RS22435* | 2.18 | *XOO_RS13790* | 2.59 | Up |
|  | *BGLU_RS22565* | 4.34 | *XOO_RS14045* | 2.26 | Up |
|  | *BGLU_RS22855* | -1.96 | *XOO_RS07555* | -1.38 | Down |
|  | *BGLU_RS23085* | 1.26 | *XOO_RS16335* | 1.48 | Up |
|  | *BGLU_RS23425* | 1.49 | *XOO_RS18645* | 3.56 | Up |
|  | *BGLU_RS23575* | -2.72 | *XOO_RS22360* | -1.69 | Down |
|  | *BGLU_RS23635* | 2.46 | *XOO_RS14055* | 2.11 | Up |
|  | *BGLU_RS23680* | 6.89 | *XOO_RS14045* | 2.26 | Up |
|  | *BGLU_RS23755* | 1.78 | *XOO_RS02315* | 2.21 | Up |
|  | *BGLU_RS24045* | 1.52 | *XOO_RS03105* | 1.73 | Up |
|  | *BGLU_RS24910* | 1.52 | *XOO_RS08880* | 2.69 | Up |
|  | *BGLU_RS25335* | -1.08 | *XOO_RS22400* | -4.83 | Down |
|  | *BGLU_RS25365* | 4.93 | *XOO_RS14045* | 2.26 | Up |
|  | *BGLU_RS26035* | 1.26 | *XOO_RS12475* | 2.26 | Up |
|  | *BGLU_RS26235* | 2.39 | *XOO_RS14045* | 2.26 | Up |
|  | *BGLU_RS26680* | 1.15 | *XOO_RS14045* | 2.26 | Up |
|  | *BGLU_RS26900* | -1.03 | *XOO_RS20145* | -1.66 | Down |
|  | *BGLU_RS26980* | -1.10 | *XOO_RS10955* | -1.03 | Down |
|  | *BGLU_RS27030* | 1.11 | *XOO_RS04240* | 3.54 | Up |
|  | *BGLU_RS27285* | -3.68 | *XOO_RS21205* | -2.28 | Down |
|  | *BGLU_RS27290* | -4.33 | *XOO_RS21210* | -2.20 | Down |
|  | *BGLU_RS27430* | -1.28 | *XOO_RS00130* | -3.17 | Down |
|  | *BGLU_RS28060* | 1.77 | *XOO_RS14035* | 1.46 | Up |
|  | *BGLU_RS28110* | 1.27 | *XOO_RS20815* | 1.72 | Up |
|  | *BGLU_RS28170* | 4.50 | *XOO_RS14045* | 2.26 | Up |
|  | *BGLU_RS28435* | 1.17 | *XOO_RS04315* | 1.84 | Up |
|  | *BGLU_RS28720* | 2.89 | *XOO_RS07025* | 1.30 | Up |
|  | *BGLU_RS28725* | 1.62 | *XOO_RS07030* | 1.96 | Up |
|  | *BGLU_RS29260* | 4.00 | *XOO_RS14045* | 2.26 | Up |
|  | *BGLU_RS29350* | 2.60 | *XOO_RS14035* | 1.46 | Up |
|  | *BGLU_RS29715* | 3.92 | *XOO_RS14045* | 2.26 | Up |

^a^Relative expression presented as log_2_(*in planta*/*in vitro*).

^b^Expression tendency of common DEGs in *in planta* condition.**Table S5. List of co-upregulated DEGs associated with MCPs and transcriptional regulators**

| Gene locus ID^a^ | Description | Degree of *in planta* gene expression^b^ | | |
| --- | --- | --- | --- | --- |
|  |  | *B. glumae* | *R. solanacearum* | *Xoo* |
| *BGLU_RS00875* | Methyl-accepting chemotaxis protein | ++ | ++ | ++ |
| *BGLU_RS02045* | Transcriptional regulator, TetR family | ++ | ++ | − |
| *BGLU_RS11100* | Methyl-accepting chemotaxis sensory transducer | ++ | + | ++ |
| *BGLU_RS11395* | Methyl-accepting chemotaxis protein | ++ | − | ++ |
| *BGLU_RS11420* | Methyl-accepting chemotaxis sensory transducer | ++ | − | ++ |
| *BGLU_RS18480* | Methyl-accepting chemotaxis sensory transducer | ++ | ++ | ++ |
| *BGLU_RS18510* | Methyl-accepting chemotaxis sensory transducer | ++ | + | ++ |
| *BGLU_RS18795* | LuxR family transcriptional regulator | ++ | ++ | − |
| *BGLU_RS18935* | Methyl-accepting chemotaxis sensory transducer | ++ | + | ++ |
| *BGLU_RS20095* | Methyl-accepting chemotaxis sensory transducer | ++ | ++ | ++ |
| *BGLU_RS20775* | Transcriptional regulator, AraC family | ++ | ++ | −− |
| *BGLU_RS22280* | Methyl-accepting chemotaxis sensory transducer | ++ | − | ++ |
| *BGLU_RS22565* | Methyl-accepting chemotaxis sensory transducer | ++ | + | ++ |
| *BGLU_RS23635* | Methyl-accepting chemotaxis protein | ++ | + | ++ |
| *BGLU_RS23680* | Methyl-accepting chemotaxis sensory transducer | ++ | + | ++ |
| *BGLU_RS23845* | RNA polymerase sigma factor FecI | ++ | ++ | + |
| *BGLU_RS23955* | Two component LuxR family transcriptional regulator | ++ | ++ | − |
| *BGLU_RS25365* | Methyl-accepting chemotaxis sensory transducer | ++ | + | ++ |
| *BGLU_RS26235* | Methyl-accepting chemotaxis sensory transducer | ++ | ++ | ++ |
| *BGLU_RS26680* | Methyl-accepting chemotaxis sensory transducer | ++ | − | ++ |
| *BGLU_RS27745* | Two component transcriptional regulator, LuxR family | ++ | ++ | − |
| *BGLU_RS28060* | Methyl-accepting chemotaxis protein | ++ | + | ++ |
| *BGLU_RS28170* | Methyl-accepting chemotaxis sensory transducer | ++ | − | ++ |
| *BGLU_RS29260* | Methyl-accepting chemotaxis sensory transducer | ++ | + | ++ |
| *BGLU_RS29350* | Methyl-accepting chemotaxis sensory transducer | ++ | − | ++ |
| *BGLU_RS29715* | Methyl-accepting chemotaxis sensory transducer | ++ | + | ++ |

^a^Gene locus ID in the reference *B. glumae* genome.

^b^++, upregulated DEG; +, upregulated gene; −−, downregulated DEG; −, downregulated gene.**Table S6. List of co-downregulated DEGs in the core genome**

| Description | Gene locus ID (Relative expression^a^) | | |
| --- | --- | --- | --- |
|  | *B. glumae* | *R. solanacearum* | *Xoo* |
| Flagellar motor protein MotB | *BGLU_RS01985* (-1.20) | *RS_RS20760*  (-1.70) | *XOO_RS17385*  (-1.06) |
| Glycine dehydrogenase | *BGLU_RS00470*  (-2.19) | *RS_RS16510*  (-2.85) | *XOO_RS17560*  (-1.07) |
| Type VI secretion protein | *BGLU_RS01965*  (-1.88) | *RS_RS20690*  (-1.69) | *XOO_RS17170*  (-1.09) |
| Fumarylacetoacetate hydrolase | *BGLU_RS16865*  (-1.96) | *RS_RS01480*  (-1.20) | *XOO_RS03740*  (-1.14) |
| N5-carboxyamino imidazole ribonucleotide mutase | *BGLU_RS15590*  (-1.25) | *RS_RS02880*  (-1.19) | *XOO_RS15885*  (-1.42) |
| Fumarate hydratase | *BGLU_RS13400*  (-1.42) | *RS_RS09830*  (-1.46) | *XOO_RS11505*  (-1.56) |
| Transketolase | *BGLU_RS02685*  (-1.20) | *RS_RS13760*  (-1.35) | *XOO_RS05685*  (-1.57) |
| DNA mismatch repair protein MutS | *BGLU_RS12840*  (-1.10) | *RS_RS05735*  (-1.26) | *XOO_RS08010*  (-1.57) |
| Heat-shock protein Hsp90 | *BGLU_RS14135*  (-1.91) | *RS_RS04900*  (-1.81) | *XOO_RS12340*  (-1.86) |
| Acetyl-CoA carboxylase Biotin carboxylase subunit | *BGLU_RS02540*  (-1.50) | *RS_RS13940*  (-1.52) | *XOO_RS02685*  (-2.00) |
| 2-oxoglutarate dehydrogenase E1 | *BGLU_RS09965*  (-1.55) | *RS_RS06360*  (-1.90) | *XOO_RS09965*  (-2.02) |
| Fructose 1,6-bisphosphatase | *BGLU_RS04195*  (-1.47) | *RS_RS10665*  (-1.33) | *XOO_RS00080*  (-2.05) |
| Enolase | *BGLU_RS12930*  (-1.22) | *RS_RS05620*  (-1.06) | *XOO_RS14640*  (-2.15) |
| Glycine cleavage system protein T | *BGLU_RS00480*  (-1.44) | *RS_RS16500*  (-3.02) | *XOO_RS08810*  (-2.22) |
| Dihydrolipoamide succinyltransferase | *BGLU_RS09960*  (-1.44) | *RS_RS06365*  (-1.52) | *XOO_RS09960*  (-2.30) |
| F0F1 ATP synthase subunit beta | *BGLU_RS00355*  (-2.20) | *RS_RS16620*  (-1.06) | *XOO_RS03550*  (-2.35) |
| Dihydrolipoamide dehydrogenase | *BGLU_RS09955*  (-1.41) | *RS_RS06370*  (-1.91) | *XOO_RS09955*  (-2.50) |
| ATPase | *BGLU_RS01975*  (-1.13) | *RS_RS20700*  (-1.58) | *XOO_RS17160*  (-2.60) |

^a^Relative expression level presented as log_2_(*in planta*/*in vitro*).

**Table S7. Bacterial strains and plasmids used in this study**

| Strain or plasmid | Description^a^ | References |
| --- | --- | --- |
| *B. glumae* |  |  |
| BGR1 | A wild type strain isolated from diseased rice in Korea, Rif^r^ | (Jeong et al., 2003) |
| BGS2 | BGR1 *tofI*::Ω | (Kim et al., 2004) |
| WBI00925 | A derivative of BGR1, *BGLU_RS00925*::pBIRS00925 | This study |
| WBI16715 | A derivative of BGR1, *BGLU_RS16715*::pBIRS16715 | This study |
| WBI23755 | A derivative of BGR1, *BGLU_RS23755*::pBIRS23755 | This study |
| WBI28435 | A derivative of BGR1, *BGLU_RS28435*::pBIRS28435 | This study |
| MBC23755 | A complement strain of WBI23755 | This study |
| *R. solanacearum* |  |  |
| GMI1000 | A wild type strain isolated from diseased tomato in French Guiana, Race 1, phylotype I | (Boucher et al., 1985) |
| WRI07255 | A derivative of GMI1000, *RS_RS07255*::Km^r^ gene cassette | This study |
| *Xoo* |  |  |
| KACC10331 | A wild type strain isolated from diseased rice in Korea, Cp^r^ | (Lee et al., 2005) |
| WXD02315 | A derivative of KACC10331, markerless deletion in *XOO_RS02315* | This study |
| MXC02315 | A complement strain of WXD02315 | This study |
| *E. coli* |  |  |
| DH5α | *E. coli* used for cloning and propagation of plasmids | Promega |
| DH5α λ*pir* | A derivative of DH5α, λ*pir* enables *ori*R6K vectors replication | (Choi et al., 2006) |
| S17-1 λ*pir* | A derivative of S17-1, RK2 *tra* regulon, λ*pir* enables *ori*R6K vectors replication | (Choi et al., 2006) |
| HB101 | *E. coli* used for triparental mating with *R. solanacearum* | Promega |
| Plasmids |  |  |
| pGEM-T Easy | A TA cloning vector, Amp^r^ | Promega |
| pVIK112 | A R6K suicide vector, *lacZY* for transcriptional fusions, Km^r^ | (Kalogeraki and Winans, 1997) |
| pBIRS00925 | A pVIK112 vector containing internal region of *BGLU_RS00925* | This study |
| pBIRS16715 | A pVIK112 vector containing internal region of *BGLU_RS16715* | This study |
| pBIRS23755 | A pVIK112 vector containing internal region of *BGLU_RS23755* | This study |
| pBIRS28435 | A pVIK112 vector containing internal region of *BGLU_RS28435* | This study |
| pRK415 | A RK-2 derived cloning vector, Tc^r^ | (Keen et al., 1988) |
| pMKm2 | A vector carrying a Km^r^ gene cassette, Amp^r^, Km^r^ | (Um et al., 2011) |
| pRIRS07255 | A pRK415 vector containing 2.0-kb PCR fragment with *RS_RS07255*::Km^r^ | This study |
| pRK2013 | A mobilization helper vector for triparental mating, Km^r^ | (Figurski and Helinski, 1979) |
| pK18*mobsacB* | A pBR322 suicide vector, *sacB*, Km^r^ | (Schäfer et al., 1994) |
| pXDRS02315 | A pK18*mobsacB* vector containing 532 bp up/downstream PCR fragment of *XOO_RS02315* | This study |
| pBBR1MCS2 | A broad-host-range vector, Km^r^ | (Kovach et al., 1995) |
| pBCRS23755 | A pRK415 containing PCR fragment of *BGLU_RS23755* | This study |
| pXCRS02315 | A pBBR1MCS2 containing PCR fragment of *XOO_RS02315* | This study |

^a^Rif^r^, rifampin resistance; Km^r^, kanamycin resistance; Cp^r^, cephalexin resistance; Amp^r^, ampicillin resistance; Tc^r^, tetracyclin resistance.

**Table S8. PCR primers used in this study**

| Primer name | Sequence (5’→3’)^a^ | Purpose |
| --- | --- | --- |
| BIRS00925_E | (*Eco*RI) GAATTCGATCTGCTGTTCACGTTCAAC | To amplify internal region of *BGLU_RS00925* |
| BIRS00925_K | (*Kpn*I) GGTACCGATCACCGTGAAGTTGATGATC |  |
| BIRS16715_E | (*Eco*RI) GAATTCCGATCAGCATCTCTTCCTTGAT | To amplify internal region of *BGLU_RS16715* |
| BIRS16715_K | (*Kpn*I) GGTACCATCGTCCACATCATCACCAAG |  |
| BIRS23755_E | (*Eco*RI) GAATTCCAACGCGATGAAGAACGG | To amplify internal region of *BGLU_RS23755* |
| BIRS23755_K | (*Kpn*I) GGTACCAGAAATGAATATGGTTCGGGC |  |
| BIRS28435_E | (*Eco*RI) GAATTCCTTGATGCGATCGACCATCT | To amplify internal region of *BGLU_RS28435* |
| BIRS28435_K | (*Kpn*I)  GGTACCCCATGGAAGACGTGCTGAT |  |
| BIRS00925_UpF | GGCGCACGTAACGAAGGAGAT | Sequencing for mutant WBI00925 |
| BIRS16715_UpF | ATGTAGATGTTGGTGCCGTCCA | Sequencing for mutant WBI16715 |
| BIRS23755_UpF | GTTCATCCCGACTACCGCTCG | Sequencing for mutant WBI23755 |
| BIRS28435_UpF | TAGTCGTCGAGCGTCTTCATGG | Sequencing for mutant WBI28435 |
| Lacfuse | GGGGATGTGCTGCAAGGCG | Sequencing for cloning pVIK112 vector and *B. glumae* mutants |
| RIRS07255_UpF | CCGACCACCAGATCCAGTT | To amplify upstream region of *RS_RS07255* |
| RIRS07255_UpR | CAGAGATTTTGAGACACACTGAAGCCACTGTATAGCGG |  |
| RIRS07255_DownF | CTAATCAGAATTGGTTAAAATCCCTGGAACGCACCC | To amplify downstream region of *RS_RS07255* |
| RIRS07255_DownR | CTCGTCGCTGAAGTACACG |  |
| Km^r^_F | TGTGTCTCAAAATCTCTG | To amplify kanamycin resistance gene cassette |
| Km^r^_R | TTAACCAATTCTGATTAG |  |
| XDRS02315_UpE | (*Eco*RI) GAATTCACGATGCCGCACACGTCAATG | To amplify upstream region of *XOO_RS02315* |
| XDRS02315_UpB | (*Bam*HI) GGATCCCATCTTGAATCTCCGTCGGCA |  |
| XDRS02315_DownB | (*Bam*HI) GGATCCTGACACGATGAGCCTGCATG | To amplify downstream region of *XOO_RS02315* |
| XDRS02315_DownH | (*Hind*III) AAGCTTGGCCACAACCATCGAAGCTG |  |
| XDRS02315_UpF | AATGCCTCCGGTCTCAACGAT | Sequencing for mutant WXD02315 |
| XDRS02315_DownR | CAACAGGCCACGCATGAAGA |  |
| pK18_DownR | GTGAAGCTAGCTTATCGCCAT | Sequencing for first-crossover recombinant |
| BCRS23755_H | (*Hind*III)  AAAAAAAGCTTAATGGACGATTCCATCCTCAGC | To amplify *BGLU_RS23755* for complementation |
| BCRS23755_B | (*Bam*HI)  AAAGGATCCTCAGCGCTCCATCGGGGT |  |
| XCRS02315_E | (*Eco*RI)  AAAAAGAATTCAATGCGTGATCCCACCTCTGA | To amplify *XOO_RS02315* for complementation |
| XCRS02315_B | (*Bam*HI)  AAAGGATCCTCACTGCCAGACCGTTTGCT |  |
| ToxA_qPCR_F | CAAGGTGGTCGGCGTGGATATC | To measure the expression change of *BGLU_RS21055* by qPCR |
| ToxA_qPCR_R | CACCACCTTGAACATCTTGCG |  |
| ToxJ_qPCR_F | GATCCGTGAACGTAGAGTC | To measure the expression change of *BGLU_RS21020* by qPCR |
| ToxJ_qPCR_R | TGTTCATGTGGATCAGCTG |  |
| ToxR_qPCR_F | CATCTGCGCGACATCTTCAAC | To measure the expression change of *BGLU_RS21050* by qPCR |
| ToxR_qPCR_R | CGGACATCGACACGCGAAACAC |  |
| TofI_qPCR_F | CTGGGTTCGTACCGTTATCG | To measure the expression change of *BGLU_RS24865* by qPCR |
| TofI_qPCR_R | GAACACTTCCTGCAACAGGTAG |  |
| TofR_qPCR_F | CTTCGAGTACTGCTGCTATG | To measure the expression change of *BGLU_RS24855* by qPCR |
| TofR_qPCR_R | CAGATGATCATGTTCGGGCTC |  |
| Control_qPCR_F | TGCGAATGGGGAGATATGCG | Control gene for normalization of qPCR |
| Control_qPCR_R | AACGTGACCCCGATCAACTG |  |

^a^Restriction enzymes are indicated in the parentheses.

**Table S9. Distribution of PCD gene in plant pathogens**

| Species | NCBI  Taxonomy ID | Phylum | Accession | Identity^a^  (%) | Coverage^b^  (%) |
| --- | --- | --- | --- | --- | --- |
| *Acidovorax citrulli* | 80869 | Proteobacteria | - | - | - |
| *Agrobacterium tumefaciens* | 358 | Proteobacteria | KIQ04082.1 | 63.87 | 96 |
| *Allorhizobium vitis* | 373 | Proteobacteria | MBB4955205.1 | 65.55 | 96 |
| *Bacillus pumilus* | 1408 | Firmicutes | - | - | - |
| *Burkholderia gladioli* | 28095 | Proteobacteria | WP_013690224.1 | 94.02 | 100 |
| *Burkholderia glumae* | 337 | Proteobacteria | WP_012733977.1 | 100 | 100 |
| *Burkholderia plantarii* | 41899 | Proteobacteria | WP_042627860.1 | 95.3 | 100 |
| *Candidatus liberibacter asiaticus* | 34021 | Proteobacteria | - | - | - |
| *Clavibacter michiganensis* | 28447 | Actinomycetota | WP_104280413.1 | 54.5 | 93 |
| *Dickeya dadantii* | 204038 | Proteobacteria | - | - | - |
| *Erwinia amylovora* | 552 | Proteobacteria | - | - | - |
| *Erwinia pyrifoliae* | 79967 | Proteobacteria | - | - | - |
| *Pantoea agglomerans* | 549 | Proteobacteria | WP_069027011.1 | 73.73 | 95 |
| *Pantoea ananatis* | 553 | Proteobacteria | WP_024471045.1 | 74.15 | 95 |
| *Pectobacterium carotovorum* subsp.*carotovorum* | 555 | Proteobacteria | WP_039507206.1 | 30.82 | 57 |
| *Pectobacterium wasabiae* | 55208 | Proteobacteria | - | - | - |
| *Pseudomonas amygdali* | 47877 | Proteobacteria | WP_003373348.1 | 62.34 | 93 |
| *Pseudomonas fuscovaginae* | 50340 | Proteobacteria | WP_010448477.1 | 60.68 | 95 |
| *Pseudomonas savastanoi* | 29438 | Proteobacteria | WP_122395308.1 | 60.85 | 95 |
| *Pseudomonas syringae*pv. *syringae* | 321 | Proteobacteria | WP_024673659.1 | 61.47 | 93 |
| *Pseudomonas viridiflava* | 33069 | Proteobacteria | WP_043192756.1 | 62.23 | 94 |
| *Ralstonia solanacearum* | 305 | Proteobacteria | WP_011001390.1 | 100 | 100 |
| *Robbsia andropogonis* | 28092 | Proteobacteria | WP_024904575.1 | 74.68 | 99 |
| *Streptomyces scabiei* | 1930 | Actinomycetota | WP_059082833.1 | 56.22 | 90 |
| *Xanthomonas axonopodis* pv. *manihotis* | 43353 | Proteobacteria | WP_017158879.1 | 91.02 | 100 |
| *Xanthomonas campestris* pv. *campestris* | 340 | Proteobacteria | WP_080639980.1 | 84.91 | 94 |
| *Xanthomonas citri* pv. *citri* | 611301 | Proteobacteria | WP_011050271.1 | 92.24 | 100 |
| *Xanthomonas oryzae* pv. *oryzae* | 64187 | Proteobacteria | WP_011257410.1 | 100 | 100 |
| *Xanthomonas translucens* | 343 | Proteobacteria | MBB4128399.1 | 84.78 | 93 |
| *Xylella fastidiosa* | 2371 | Proteobacteria | - | - | - |

^a^BLASTp identity against PCD genes.

^b^BLASTp coverage against PCD genes.

**REFERENCES**

**Boucher, C., Barberis, P., Trigalet, A., and Demery, D.** (1985). Transposon mutagenesis of *Pseudomonas solanacearum*: Isolation of Tn5-induced avirulent mutants. Microbiology **131:** 2449–2457.

**Cai, Z., Yuan, Z.H., Zhang, H., Pan, Y., Wu, Y., Tian, X.Q., Wang, F.F., Wang, L., and Qian, W.** (2017). Fatty acid DSF binds and allosterically activates histidine kinase RpfC of phytopathogenic bacterium *Xanthomonas campestris* pv. *campestris* to regulate quorum-sensing and virulence. PLoS Pathog. **13:** e1006304.

**Choi, K.H., DeShazer, D., and Schweizer, H.P.** (2006). mini-Tn7 insertion in bacteria with multiple *glmS*-linked *att*Tn7 sites: example *Burkholderia mallei* ATCC 23344. Nat. Protoc. **1:** 162–169.

**Figurski, D., and Helinski, D.** (1979). Replication of an origin-containing derivative of plasmid RK2 dependent on a plasmid function provided in *trans*. Proc. Natl. Acad. Sci. U. S. A. **76:** 1648–1652.

**Jacobs, J.M., Babujee, L., Meng, F., Milling, A., and Allen, C.** (2012). The *in planta* transcriptome of *Ralstonia solanacearum*: conserved physiological and virulence strategies during bacterial wilt of tomato. MBio **3:** e00114-12.

**Jeong, Y., Kim, J., Kim, S., Kang, Y., Nagamatsu, T., and Hwang, I.** (2003). Toxoflavin produced by *Burkholderia glumae* causing rice grain rot is responsible for inducing bacterial wilt in many field crops. Plant Dis. **87:** 890–895.

**Kalogeraki, V.S., and Winans, S.C.** (1997). Suicide plasmids containing promoterless reporter genes can simultaneously disrupt and create fusions to target genes of diverse bacteria. Gene **188:** 69–75.

**Keen, N., Tamaki, S., Kobayashi, D., and Trollinger, D.** (1988). Improved broad-host-range plasmids for DNA cloning in gram-negative bacteria. Gene **70:** 191–197.

**Kim, J., Kim, J.G., Kang, Y., Jang, J.Y., Jog, G.J., Lim, J.Y., Kim, S., Suga, H., Nagamatsu, T., and Hwang, I.** (2004). Quorum sensing and the LysR-type transcriptional activator ToxR regulate toxoflavin biosynthesis and transport in *Burkholderia glumae*. Mol. Microbiol. **54:** 921–934.

**Kim, S., Park, J., Lee, J., Shin, D., Park, D.S., Lim, J.S., Choi, I.Y., and Seo, Y.S.** (2014). Understanding pathogenic *Burkholderia glumae* metabolic and signaling pathways within rice tissues through *in vivo* transcriptome analyses. Gene **547:** 77–85.

**Kovach, M.E., Elzer, P.H., Steven Hill, D., Robertson, G.T., Farris, M.A., Roop, R.M., and Peterson, K.M.** (1995). Four new derivatives of the broad-host-range cloning vector pBBR1MCS, carrying different antibiotic-resistance cassettes. Gene **166:** 175–176.

**Lee, B.M., Park, Y.J., Park, D.S., Kang, H.W., Kim, J.G., Song, E.S., Park, I.C., Yoon, U.H., Hahn, J.H., Koo, B.S., Lee, G.B., Kim, H., Park, H.S., Yoon, K.O., Kim, J.H., Jung, C. hee, Koh, N.H., Seo, J.S., and Go, S.J.** (2005). The genome sequence of *Xanthomonas oryzae* pathovar *oryzae* KACC10331, the bacterial blight pathogen of rice. Nucleic Acids Res. **33:** 577–586.

**Lee, S.W., Glickmann, E., and Cooksey, D.A.** (2001). Chromosomal locus for cadmium resistance in *Pseudomonas putida* consisting of a cadmium-transporting ATPase and a MerR family response regulator. Appl. Environ. Microbiol. **67:** 1437–1444.

**Lim, J., Lee, T., Nahm, B., Choi, Y., Kim, M., and Hwang, I.** (2009). Complete genome sequence of *Burkholderia glumae* BGR1. J. Bacteriol. **191:** 3758–3759.

**Luo, Z.Q., and Farrand, S.K.** (1999). Signal-dependent DNA binding and functional domains of the quorum-sensing activator TraR as identified by repressor activity. Proc. Natl. Acad. Sci. U. S. A. **96:** 9009–9014.

**Mac Fhionnlaoich, N., Ibsen, S., Serrano, L.A., Taylor, A., Qi, R., and Guldin, S.** (2018). A toolkit to quantify target compounds in thin-layer-chromatography experiments. J. Chem. Educ. **95**: 2191–2196.

**Qureshi, R., and Sacan, A.** (2013). Weighted set enrichment of gene expression data. BMC Syst. Biol. **7:** S10.

**Salanoubat, M., Genin, S., Artiguenave, F., Gouzy, J., Mangenot, S., Arlat, M., Billault, A., Brottier, P., Camus, J.C., Cattolico, L., Chandler, M., Choisne, N., Claudel-Renard, C., Cunnac, S., Demange, N., Gaspin, C., Lavie, M., Moisan, A., Robert, C., Saurin, W., Schiex, T., Siguier, P., Thébault, P., Whalen, M., Wincker, P., Levy, M., Weissenbach, J., and Boucher, C.A.** (2002). Genome sequence of the plant pathogen *Ralstonia solanacearum*. Nature **415:** 497–502.

**Sambrook, J., and Russell, D.W.** (2001). *Molecular cloning: A laboratory manual.* 3rd ed. Cold Spring Harbor Lab Press, New York, USA.

**Schäfer, A., Tauch, A., Jäger, W., Kalinowski, J., Thierbach, G., and Pühler, A.** (1994). Small mobilizable multi-purpose cloning vectors derived from the *Escherichia coli* plasmids pK18 and pK19: selection of defined deletions in the chromosome of *Corynebacterium glutamicum*. Gene **145:** 69–73.

**Um, H.Y., Chung, E., Lee, J.H., and Lee, S.W.** (2011). Improved antibiotic resistance gene cassette for marker exchange mutagenesis in *Ralstonia solanacearum* and *Burkholderia* species. J. Microbiol. **49:** 305–308.
